# Influence of Commercial Ionomers and Membranes on a PGM-Free Catalyst in the Alkaline Oxygen Reduction

**DOI:** 10.1021/acsaem.4c02929

**Published:** 2025-03-06

**Authors:** Simon Kellner, Ziyang Liu, Francesco D’Acierno, Angus Pedersen, Jesús Barrio, Sandrine Heutz, Ifan E. L. Stephens, Silvia Favero, Maria-Magdalena Titirici

**Affiliations:** † Department of Chemical Engineering, Imperial College London, London SW7 2AZ, United Kingdom; ‡ Department of Materials, Royal School of Mines, Imperial College London, London SW7 2AZ, United Kingdom; § Advanced Institute for Materials Research (WPI-AIMR), Tohoku University 2-1-1 Katahira, Aobaku, Sendai, Miyagi 980-8577, Japan

**Keywords:** alkaline exchange ionomer, alkaline exchange membrane, PGM-free catalysts, Fe−N−C catalyst, gas-diffusion electrode, oxygen reduction reaction, fuel cell

## Abstract

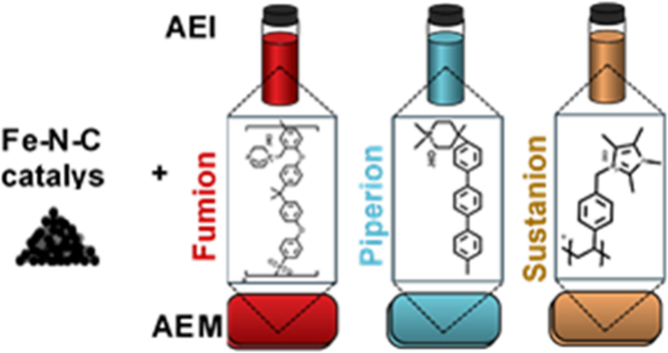

Hitherto, research into alkaline exchange membrane fuel cells lacked a commercial benchmark anionomer and membrane, analogous to Nafion in proton-exchange membrane fuel cells. Three commercial alkaline exchange ionomers (AEIs) have been scrutinized for that role in combination with a commercial platinum-group-metal-free Fe–N–C (Pajarito Powder) catalyst for the cathode. The initial rotating disc electrode benchmarking of the Fe–N–C catalyst’s oxygen reduction reaction activity using Nafion in an alkaline electrolyte seems to neglect the restricted oxygen diffusion in the AEIs and is recommended to be complemented by measurements with the same AEI as used in the alkaline exchange membrane fuel cell (AEMFC) testing. Evaluation of the catalyst layer in a gas-diffusion electrode setup offers a way to assess the performance in realistic operating conditions, without the additional complications of device-level water management. Blending of a porous Fe–N–C catalyst with different types of AEI yields catalyst layers with different pore size distributions. The catalyst layer with Piperion retains the highest proportion of the original BET surface area of the Fe–N–C catalyst. The water adsorption capacity is also influenced by the AEI, with Fumion FAA-3 and Piperion having equally high capabilities surpassing Sustainion. Finally, the choice of the membrane influences the ORR performance as well; particularly, the low hydroxide conductivity of Fumion FAA-3 in the room temperature experiments mitigates the ORR performance irrespective of the AEI in the catalyst layer. The best overall performance at high current densities is shown by the Piperion anion exchange ionomer matched with Sustainion X37–50 membrane.

## 1 Introduction

The invention of Nafion membranes, which have been developed by Walther Grot and patented by DuPont, played a transformative role in enabling the proton-exchange-membrane fuel cells (PEMFCs) technology.^1^ In 2023, the European Chemicals Agency (ECHA) proposed a ban on per- and polyfluoroalkyl substances, including Nafion, whose chemical structure is based on a perfluorinated polymer backbone functionalized with sulfonic acid groups.^2^ This potential restriction on Nafion could benefit hydrocarbon-based anion-conducting polyelectrolytes, which are cheaper and safer to produce.^3^ The progress in anion-conducting polyelectrolyte development over the past 20 years has accelerated the research on alkaline exchange membrane fuel cells (AEMFCs).^4^ Recently, platinum-group metal (PGM)-based AEMFCs achieved a breakthrough of peak power densities >2 W/cm^2^ (Table S1).^5−7^ In AEMFCs, PtRu/C is usually used as the anode benchmark catalyst with Pt loadings much higher than those at the anode in PEMFCs, while Pt/C is used at the cathode. As the oxygen reduction reaction (ORR) is more favorable with faster electrochemical kinetics in alkaline medium, the catalyst loading at the cathode can be reduced. Alternatively, the cathode PGM catalyst can be substituted by a PGM-free catalyst such as Fe–N–C catalyst materials, which are among the best-performing PGM-free catalysts in an alkaline environment.^8−12^ The only commercial catalyst among the AEMFC cathodes exceeding the peak power density of 1 W/cm^2^ is Fe–N–C Pajarito Powder (Table S2).^9,13,14^ Its synthesis and characterization have been described elsewhere.^15,16^

The critical component in AEMFCs is the anion-conducting polyelectrolyte applied as ionomer (AEI) in the catalyst layers and as membrane (AEM) between the anode and cathode.^17^ While the requirement on the anion-conducting polyelectrolyte differs depending on the application as either an AEI or an AEM, both need sufficient ion conductivity for hydroxide ion (OH^–^) transport. AEMs are positioned between the anode and cathode to block hydrogen and oxygen crossover, whereas AEIs are a component of anode and cathode catalyst layers acting as binder and supply anions to the triple-phase boundary. Therefore, AEMs require good gas barrier properties^18^ and limited water contents to minimize membrane swelling, while AEIs need high water permeability, fast oxygen transport, and minimal blocking of the electrocatalysts’ active sites. Ideally, the development of AEIs and AEMs should take place separately to design the properties of the material for the specific function.^19^ We refer to recent reviews covering the materials development for AEIs^20^ and for AEMs.^21^ The rapid development led to the scale-up and commercialization of anion-conducting polyelectrolyte technologies, such as those offered by FUMATECH BWT GmbH, Versogen Inc. and Dioxide Materials Inc.

Gas-diffusion electrodes (GDEs) offer an intermediate opportunity between rotating disk electrode measurements and membrane electrode assembly (MEA) to characterize the application-relevant catalyst layer.^22^ The utilization of the catalyst can be maximized by tailoring the ionomer to catalyst ratio; the efficiency of water and O_2_ mass transport can be enhanced by fine-tuning the microstructure via the choice of solvents in the ink,^23^ and the optimal total loading of catalyst can be determined. The door toward alkaline studies in a GDE setup was opened by a study of commercial Aemion ionomer with Pajarito Powder’s Fe–N–C catalyst and focused on the activation time of the catalyst layer and catalyst durability.^24^

**Scheme 1:**
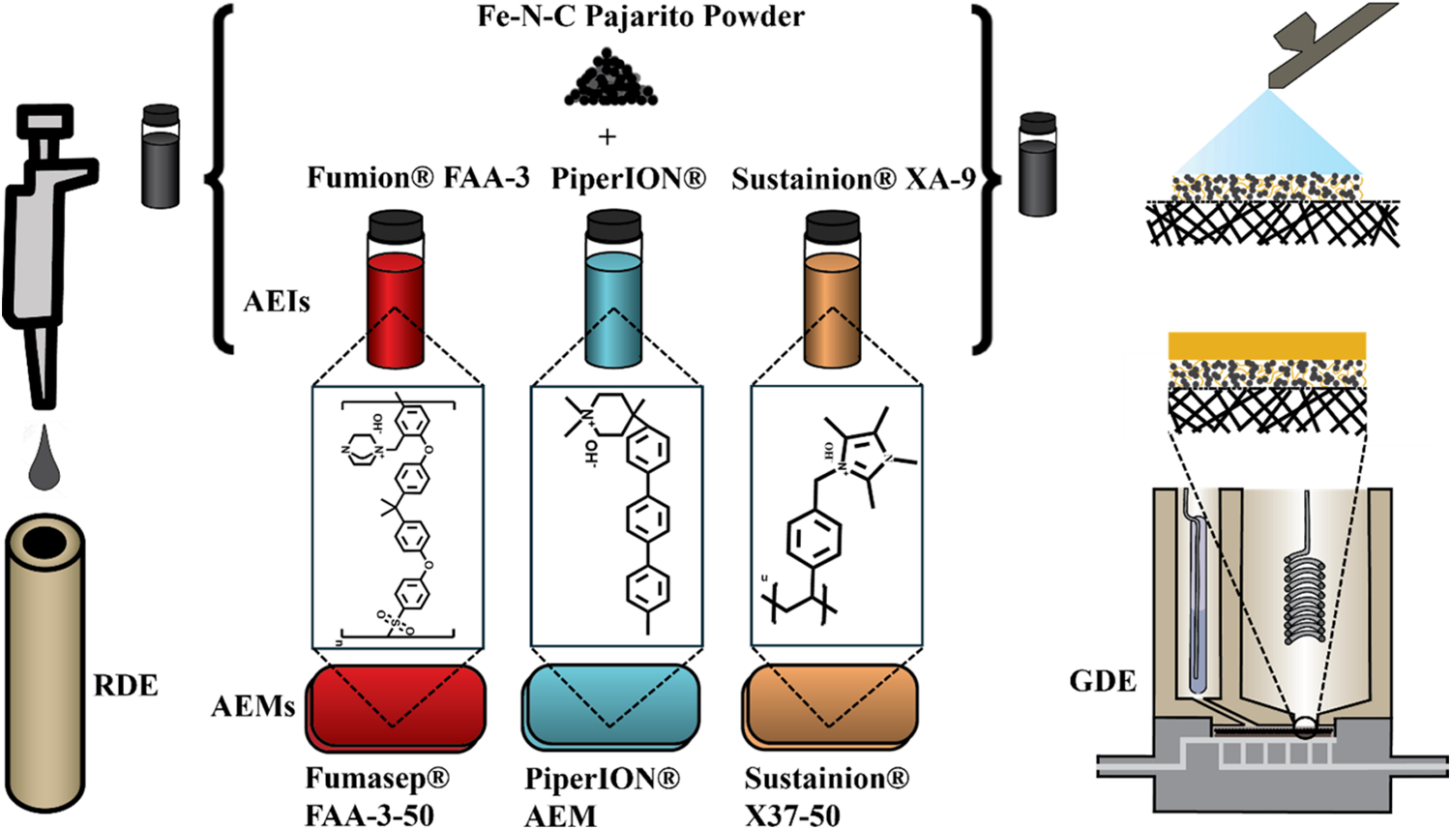
Ink Based on Commercial Fe–N–C Pajarito Powder and One of Three Types of Commercial AEI is Either Drop-Casted on the RDE or Sprayed onto the Gas-Diffusion Layer The gas-diffusion electrode is attached to a commercial AEM and tested in a gas-diffusion-electrode half-cell (molecular structures are also displayed in Figure S1).

We recently reported the performances of a biomass-derived Fe–N–C and Pajarito Powder Fe–N–C at alkaline pH in the small GDE half-cell^25^by employing Sustainion XA-9 ionomer and Sustainion’s X37–50 membrane.^26^ Following up on our previous results, in this work we present an extensive study on cathode catalyst layers based on Fe–N–C from Pajarito Powder and the three AEIs including Fumion FAA-3 (abbrev.: Fumion AEI), PiperION (abbrev.: Piperion AEI), and Sustainion XA-9 (abbrev.: Sustainion AEI), which differ in their molecular structures (Figure S1). In contrast to the Aemion study,^24^ in the GDE employed in this work, the catalyst layer is separated by a membrane from the liquid electrolyte compartment to prevent flooding of the catalyst layer and this configuration enables us to assess the effect of cross-combining the selected ionomer with the membrane equivalents of Fumasep FA-3–50 (FumaTech) (abbrev.: Fumasep AEM), PAP-TP-85 (Piperion AEM) (abbrev.: Piperion AEM), and X37–50 (Sustainion) (abbrev.: Sustainion AEM) (Scheme 1).

The effect of the ionomer interaction with Fe–N–C catalyst was characterized ex situ in a rotating disc electrode (RDE) study combined with small-angle X-ray scattering (SAXS) study. The ORR performance of the sprayed catalyst layers was assessed in a gas-diffusion-electrode cell. The most important properties of the catalyst layer were determined ex situ or in situ and correlated with the ORR performance. The catalyst morphology was characterized by SEM imaging and porosimetry analysis; the hydrophobicity of the catalyst layer was assessed by water uptake measurements, and gas-diffusion properties were determined by means of O_2_ limiting current measurements. The catalyst inks were also characterized by SAXS, rheology, and DLS to correlate their properties to those of the catalyst layers. In addition, the choice of AEM with its intrinsic hydroxide conductivity impacted the measured ORR performance.

## 2 Results and Discussion

### 2.1 Rotating Disc Electrode Study of AEIs and SAXS Measurement of RDE Inks

RDE is the most conventional method to benchmark the catalytic activity of ORR catalysts in the kinetic region. Routinely, Fe–N–C catalysts are dispersed in an ink composed of a ionomer and solvents and that ink is drop-casted on a glassy carbon electrode with a loading commonly in the range of 200–800 μg/cm^2^. Recently, Beltrán and Litster conducted a meta-analysis of RDE studies and pointed out that the metric of half-wave potential increases as the RDE catalyst loading is increased. They concluded that the mass activity is a better metric for intrinsic activity than the half-wave potential.^27^ They also recommended lower loadings, as high loadings may diminish the mass activity due to transport losses and lower accessibility to active sites. Therefore, for this work, we chose a catalyst loading of 200 μg/cm^2^. It is important to highlight that RDE studies of ionomers can provide limited information, masking the role of the ionomer in water management, its performance at low humidity, and even its anion conductivity, since in a liquid environment this property is not critical. Nevertheless, RDE can give insights into specific properties of the ionomer, including those that affect catalyst layer morphology and kinetics.

**Figure 1:**
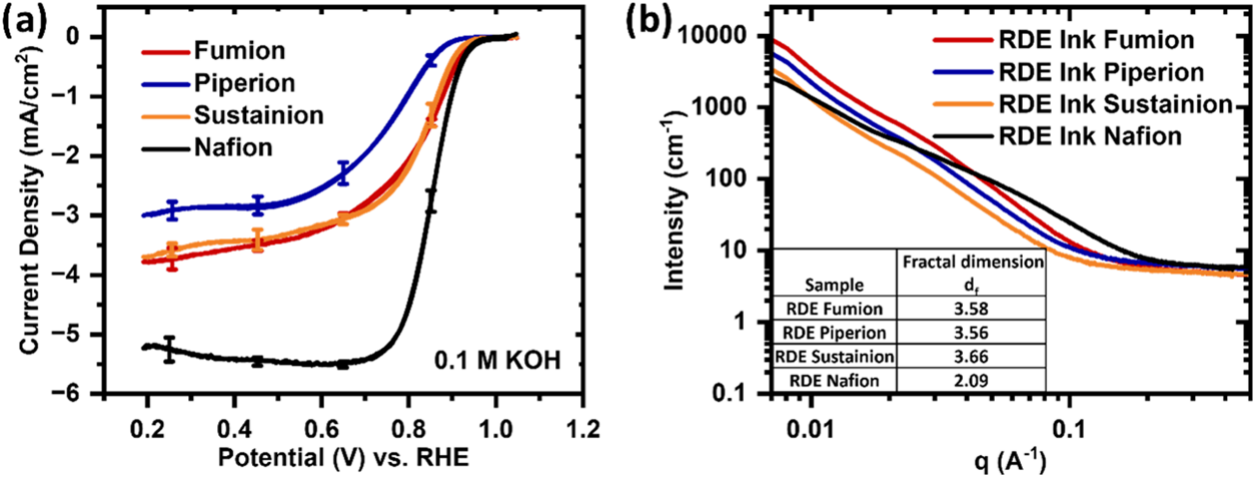
(a) Cyclic voltammetry (CV) of Fe–N–C Pajarito Powder catalyst with the respective ionomer, Fumion, Piperion, Sustainion and Nafion (33% ionomer/66% catalyst), starts at a potential of 0.20 V_RHE_ with a 10 mV/s scan rate in the anodic scan direction in oxygen-saturated 0.1 M KOH at 1600 rpm. The catalyst loading of 200 μg_Fe–N–C_/cm^2^ is measured on a glassy carbon electrode (Ø = 5 mm) (WE) in a cell containing a glassy carbon rod (CE) and Hg/HgO (RE). The applied voltage is corrected for *iR* drop determined by EIS post measurement. The average of a set of three independent measurements for each sample is plotted with error bars. (b) SAXS scattering curves of catalyst inks for RDE.

The effects of the different commercial ionomers Nafion, Fumion, Piperion, and Sustainion on the ORR performance of a commercially available Fe–N–C catalyst (Pajarito Powder) are compared (Figure 1a). Even though the catalyst nature, loading, and ink composition are kept constant for all ionomers, the cyclic voltammograms in oxygen-saturated 0.1 M KOH are quite different. In the entire potential range, the combination with Nafion has the best activity followed by that with Fumion and Sustainion, with very similar performance. The combination of the Fe–N–C catalyst with Piperion shows the worst kinetic performance. Even though Nafion’s molecular structure contains sulfonic acid groups for proton transport in an acidic electrolyte, Nafion is used as ionomer in the majority of RDE studies in alkaline electrolytes.^28,29^ Jaouen et al. and Pescarmona et al. have reported that the use of Fumion instead of Nafion in RDE leads to a lower kinetic current density with Fe–N–C and metal-free catalysts, respectively.^30,31^ The mass activities at 0.85 V are the highest with Nafion (30.35 A/g), followed by Fumion (10.90 A/g), Sustainion (9.65 A/g), and finally Piperion (2.05 A/g). The same trend can be found for the limiting current density at 0.2 V. The highest limiting current was found with Nafion (5.18 mA/cm^2^), followed by Fumion (3.78 mA/cm^2^), Sustainion (3.68 mA/cm^2^), and Piperion (2.98 mA/cm^2^). Theoretically, diffusion-limiting currents of around 5.97 mA/cm^2^ are expected at 1600 rpm on a 5 mm diameter working electrode in 0.1 M KOH.^32^ Obviously, the AEIs block the transport of oxygen to the catalyst’s active site, whereas in the case of Nafion the oxygen transport is not restricted.

The RDE inks are investigated with SAXS to gain insights into the agglomerates of the ionomer and catalyst in the ink. The scattered intensity (*I*(*q*)) is plotted versus the scattering vector (*q*) for the four different inks (Figure 1b). The power-law scaling exponent of the intensity at low *q* (<0.01 Å^–1^) in *I*(*q*) ∼*q*_f_^–d^ is called the fractal dimension *d*_f_.^33^ The fractal dimension represents the agglomerate structure, where a larger *d*_f_ indicates a higher level of agglomeration. As listed in the table, the *d*_f_ values for the AEI (3.58; 3.56; 3.66) are at least 70% larger than the one obtained for Nafion (2.09). These values suggest that the AEI inks contain larger agglomerates than the ink based on Nafion. Larger aggregates are suspected to reduce the triple-phase boundary and catalyst utilization and therefore lead to poorer performance in the kinetic region.^34^

Electrochemical impedance spectroscopy (EIS) recorded at 0.85 V in oxygen at 1600 rpm shows larger-diameter semicircles at low frequencies for the AEIs compared to Nafion (Figure S2). While we acknowledge that the interpretation of EIS is nontrivial, the lower magnitude of the low-frequency intercept on the real axis is consistent with the notion that the ionomer imposes a restriction on ion diffusivity.^35^ We propose that the origin of the lower local oxygen transport for the commercial AEIs is due to the lack of oxygenophilic perfluorocarbons, which are present in Nafion. Supporting this conclusion, Xu et al. have shown that modifying the molecular structure of an AEI by replacing methyl (−CH_3_) or trifluoromethyl groups (−CF_3_) increases the RDE limiting current density by 15% (from 4.5 to 5.3 mA/cm^2^).^36^

### 2.2 Preparation of the Catalyst Layer for Gas-Diffusion Electrode (GDE) Testing–from Ink to Morphology

Inks for GDE testing are prepared with a lower ionomer to catalyst ratio (20/80 wt % I/C) compared to RDE inks (33/66 wt % I/C) following the best-reported performance of Fe–N–C Pajarito Powder in AEMFC with ETFE-based radiation-grafted benzyltrimethylammonium-type ionomer.^13^ The GDE inks are characterized by rheology and dynamic light scattering (DLS) to gain insights into how the ink microstructure affects the catalyst layer structure. There are multiple parameters to adjust in the ink composition, including types of solvents,^37^ solvent ratio,^38^ concentration of catalyst, and concentration of ionomer.^39^ For the sake of focusing on the AEI properties, all of these parameters are kept constant and only the type of AEI is changed. Ethanol is chosen as the solvent for the GDE ink as the technical data sheets of all AEIs state solubility in ethanol^40^ or the AEI in ethanol dispersion are supplied, as it is the case for Piperion and Sustainion. In contrast to the RDE inks studied in the previous section, which can be ultrasonicated to redisperse the ink fully before drop-casting it, the dispersibility of a given catalyst in an ink is crucial in the case of a GDE. The duration of the spraying of the electrode compared to RDE drop-casting is much longer; precipitation of the ionomer or catalyst in the ink container of the spray gun should be avoided. We chose the concentration of the catalyst in the ink to be 2 mg_Cat_/mL (0.2 wt %), as higher concentrations caused frequent clogging of the spray gun needle.

**Figure 2:**
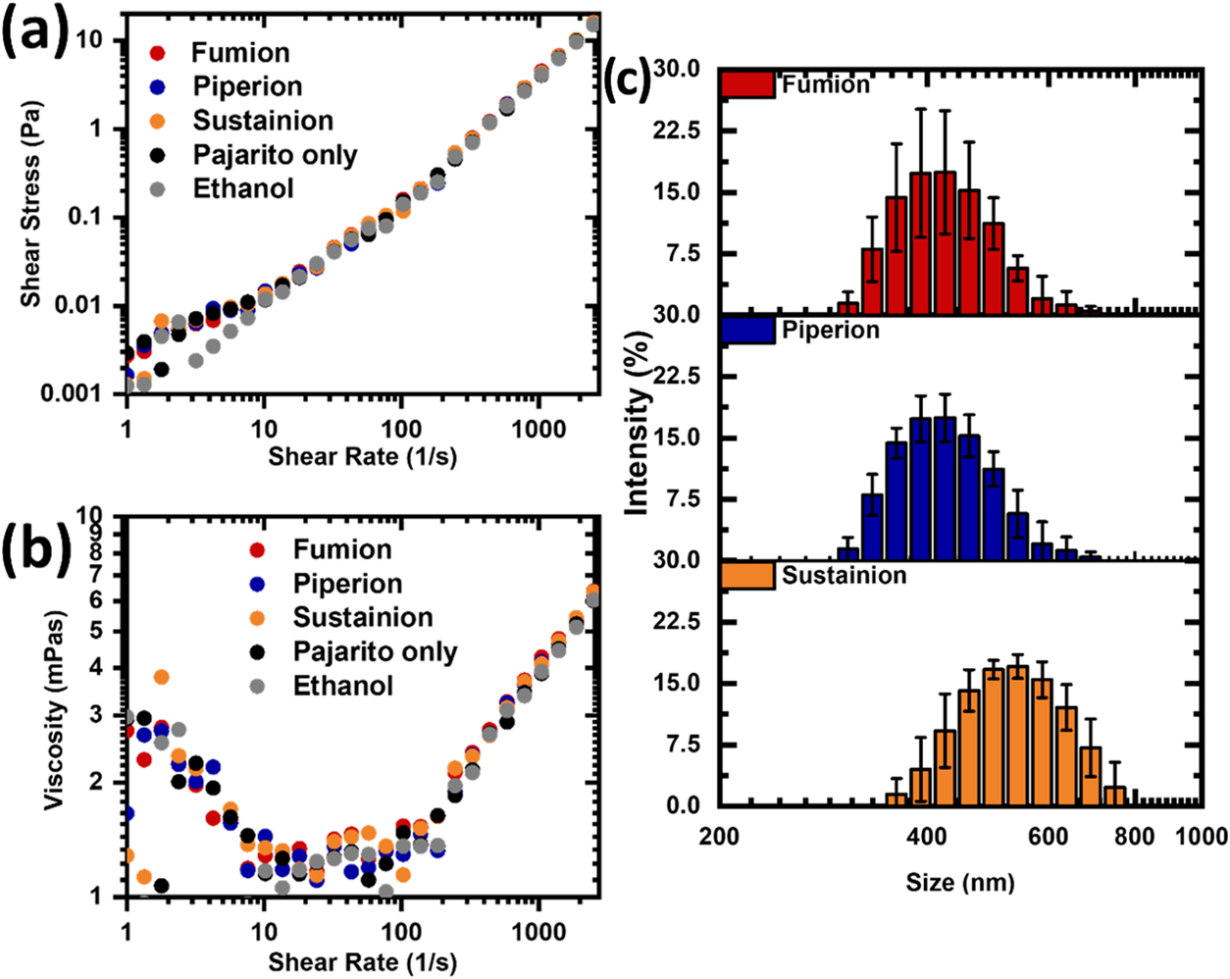
(a) Shear stress and (b) viscosity for the AEI/Fe–N–C GDE inks and Fe–N–C only and solvent ethanol only, recorded at 25 °C. (c) Aggregate size distribution of the GDE inks. The inks were diluted by a factor of 100.

The GDE inks are characterized by a rheology study (Figure 2a). The shear stress linearly increases with a constant slope for ethanol, slightly deviating from this linear increase at shear rates below 10 s^–1^ for the inks containing the AEIs and Pajarito Powder and the reference with Pajarito Powder in ethanol. The straight line observed for ethanol is typical for Newtonian fluids, such as ethanol. The presence of Pajarito Powder with and without AEIs in the inks causes weak shear thickening, as can be seen from the minor increase of the shear stress at shear rates below 10 s^–1^. The viscosity stays constant in the shear rate window from 10 to 100 s^–1^ for all inks (Figure 2). At lower and higher shear rates, the viscosity tends to increase. It can be concluded that the type of AEI does not affect the viscosity of the ink.

The aggregate sizes in the catalyst inks are determined by dynamic light scattering (DLS). The average aggregate sizes for the inks with Pajarito Powder and Fumion and Piperion AEIs are distributed between 300 and 700 nm (Figure 2c). Most aggregates for these two inks can be found between 400 and 500 nm. A shift of the aggregate distribution by 100 nm to larger aggregates is observed for the inks containing Pajarito Powder and Sustainion AEI.

**Figure 3:**
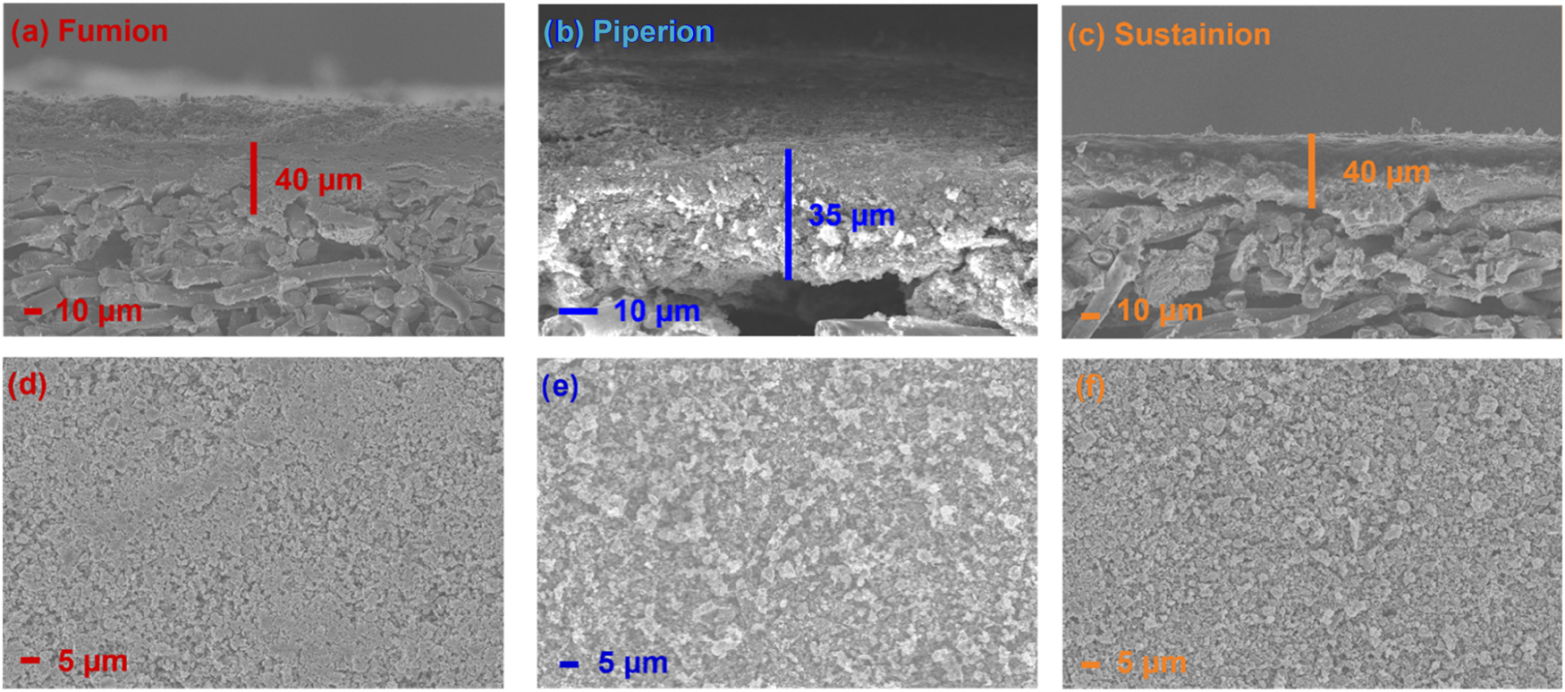
Cross section of catalyst layers based on Pajarito Powder and (a) Fumion (loading: 1.31 mg/cm^2^), (b) Piperion (loading: 1.20 mg/cm^2^), and (c) Sustainion (loading: 1.28 mg/cm^2^); the top views of catalyst layers with (d) Fumion, (e) Piperion, and (f) Sustainion before electrochemical characterization.

After spraycoating onto the hydrophobic side of the gas-diffusion layer H23C8 (Freudenberg), the morphologies of the catalyst layers are studied via scanning electron microscopy (SEM) (Figure 3). The loading of the catalyst layers is verified by cross-section imaging. The catalyst loadings were confirmed by weighing Fumion AEI as 1.31 mg_Fe–N–C_ cm^–2^, Piperion AEI as 1.20 mg_Fe–N–C_ cm^–2^, and Sustainion AEI as 1.28 mg_Fe–N–C_ cm^–2^. The cross-section images allowed the thicknesses of the films to be determined: 40 μm for Fumion AEI/Fe–N–C, 35 μm for Piperion AEI/Fe–N–C, and 40 μm for Sustainion AEI/Fe–N–C. The catalyst layers show different surface features. The surface based on Fumion AEI is smooth, with catalyst particles forming flat and dense island aggregate structures. In the Piperion AEI-containing catalyst layer, a rough surface with rather loose out-of-the-surface plane aggregates can be observed, while in the case of the Sustainion AEI-based catalyst layer, a homogeneous distribution of catalyst particles is observed.

### 2.3 Effect of the AEI Type on the Oxygen Reduction Reaction Performance of Fe–N–C Catalysts in Alkaline Gas-Diffusion Electrode

**Figure 4:**
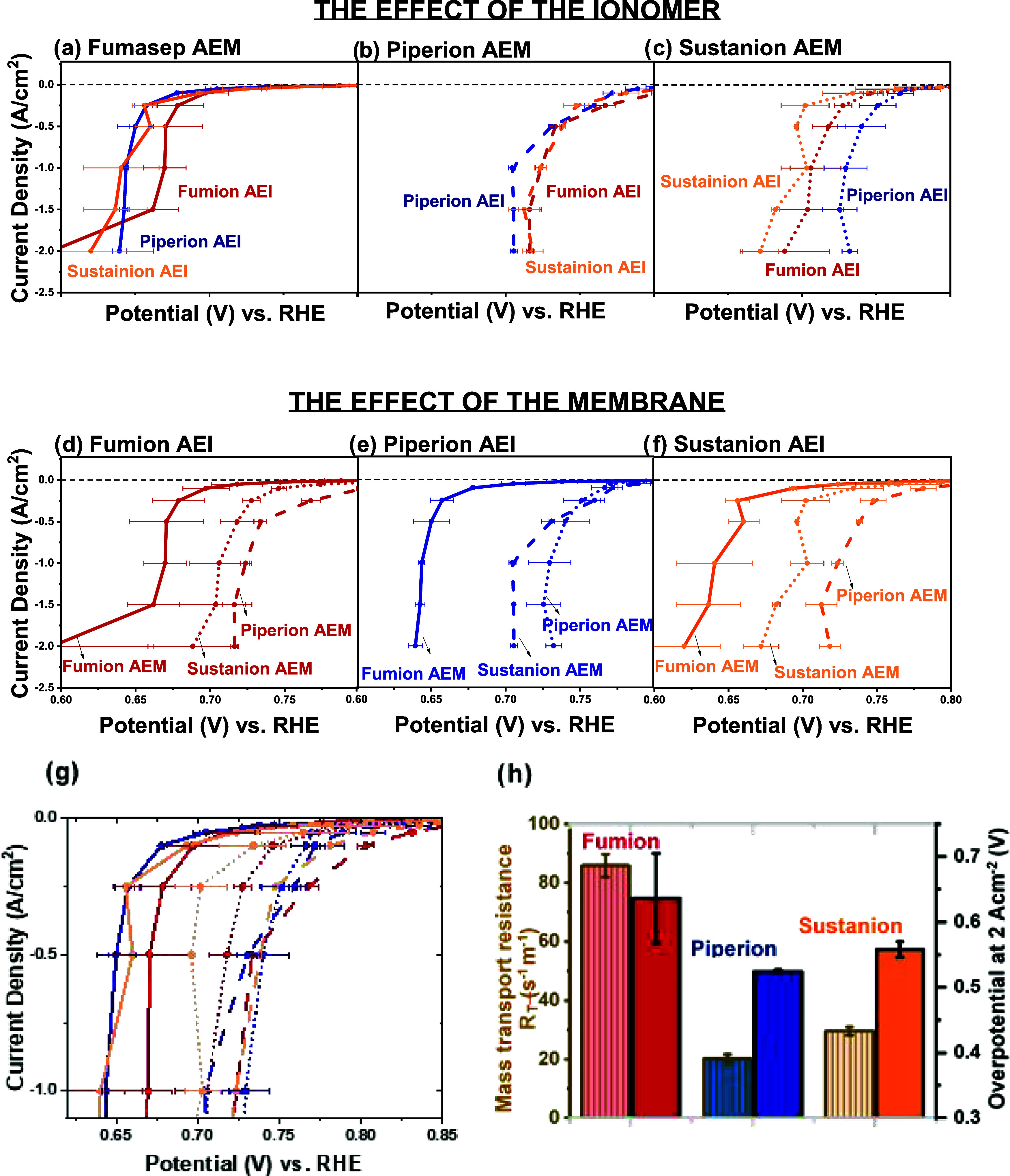
(a–f) Oxygen reduction reaction measurement with GEIS protocol. The same data are plotted in (a–f). The first three graphs show the effect of the ionomer while using the same membrane, while graphs (d–f) show the effect of changing the membrane while using the same ionomer. Data collected with the ionomer Fumion are colored red, the ionomer Piperion blue, and the ionomer Sustanion yellow. On the contrary, membranes are distinguished by the line style (solid for Fumion, long dash for Piperion, and dots for Sustanion). The GEIS protocol starts with galvanostatic steps and holding time from small current densities to high current densities in the order of −0.1 mA/cm^2^ (90 s), −1/-2.5/-5/-10 mA/cm^2^ (30 s), −25/-50/-10/-250 mA/cm^2^ (5 s), and −0.5/-1.0/-1.5/-2.0 A/cm^2^ (5 s) at an oxygen flow rate of 300 mL/min. The last potential value recorded at each galvanostatic step is taken. Each single potential value is *iR*-compensated with the value of the uncompensated resistance, corresponding to the magnitude of the impedance measured after the corresponding galvanostatic step. At least two sets of independent GEIS measurements for each type of sample are carried out. (g) Zoom-in at lower current density range. (h) Mass transport resistance *R*_T_ of the catalyst layers with different ionomers at 2.5% O_2_ (brown border) besides the potential at 2 A/cm^2^ at 100% O_2_ (black border). Details of the determination of the limiting current *i*_lim_ and mass transport resistance *R*_T_ can be found in the Supporting Information. Two independent measurements of one type of sample are used to determine the limiting current.

**Figure 5:**
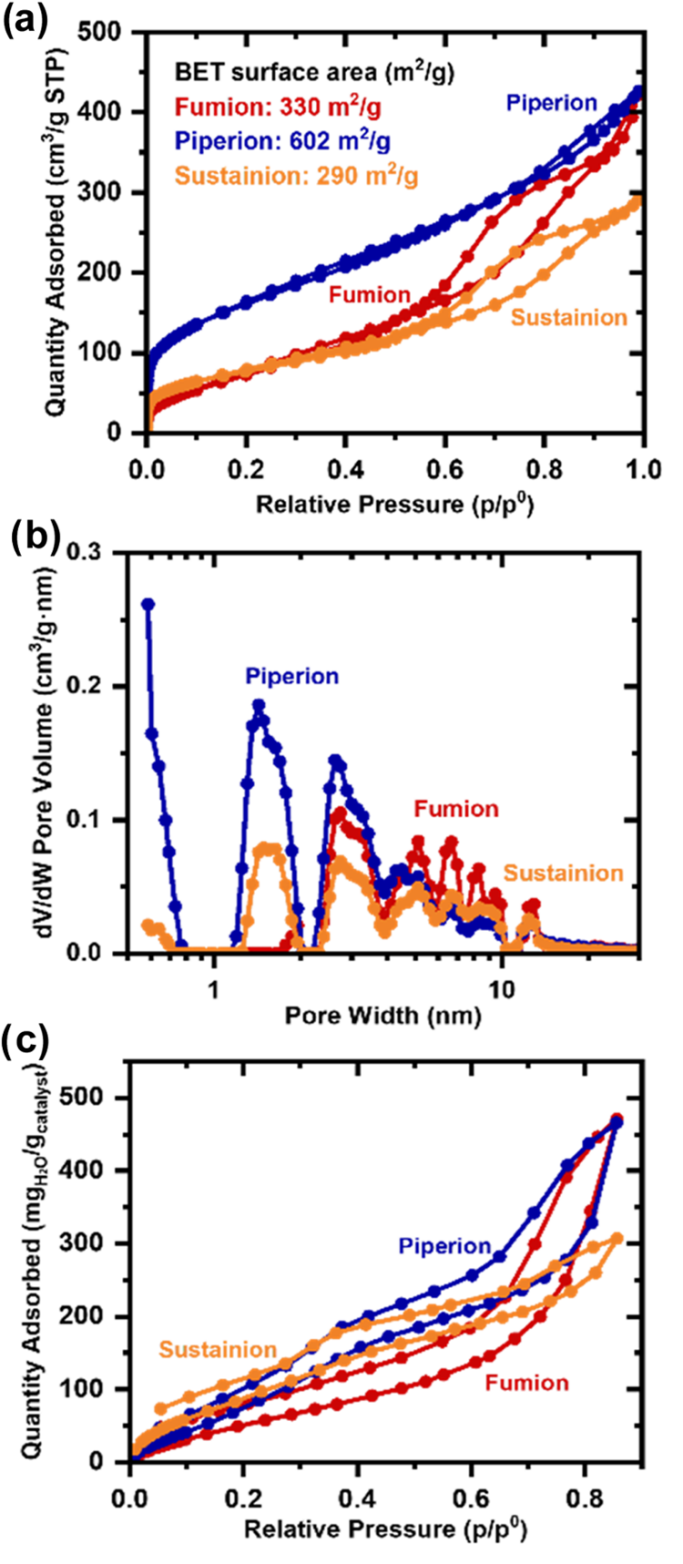
(a) Nitrogen sorption isotherm of the catalyst layers sprayed on aluminum foil. (b) Pore size distribution. (c) Water sorption isotherms before electrochemical characterization.

Figure 4 shows the oxygen reduction current obtained with Fe–N–C catalysts and the commercial anion exchange ionomer/membrane with Figure 4a–c focusing on the effect of the ionomer for a given membrane. At high current densities (2 A/cm^2^), the average combinations with Piperion AEI have a lower overpotential regardless of the membrane (Figure 4b). One potential explanation might be that Pajarito Powder retains a high Brunauer–Emmett–Teller (BET) surface area of 603 m^2^/g after integration into the catalyst layer with Piperion AEI compared to the original BET surface area of Pajarito Powder itself (675 m^2^/g) (Figure 5a). Therefore, there might be more active sites still accessible at higher current densities.

The best performance at high current densities is not achieved by a pair of the same anion-conducting polyelectrolyte family, but by Piperion AEI with Sustainion AEM. This is in agreement with the report by Lee et al., where it was observed that using the same anion-conducting polyelectrolyte as membrane and ionomer might not be the ideal solution due to different requirements for membrane and ionomer.^17^ Since Sustainion AEM was the membrane, which gave the best combination at high current densities, a combination of an unmodified Nafion ionomer with Pajarito Powder and Sustainion AEM was also tested (Figure S5). Despite Nafion molecular structure being designed for proton transport, it showed a comparable performance to Fumion AEI. Nafion has been also used as a binder in an AEMFC; thus, it can be suspected that the water channels might be responsible for the hydroxide transport in the catalyst layer at high relative humidities.^41^

The worst performance at high current densities on average is that of the combination of Fumion AEI and Fumasep AEM. Among 5 measurements, 4 showed bubble formation in the upper cell compartment opening above the membrane, potentially as a result of damage to the membrane at high current densities. It should be noted that the curves show slightly lower overpotentials for some samples at high current densities (1, 1.5, and 2.0 A/cm^2^), deviating from the slope (including Sustainion AEI/Sustainion AEM, Sustainion AEI/Piperion AEM, Piperion AEI/Sustainion AEM). These are considered time-dependent artifacts, since the recorded data are a mixture of time and potential-dependent processes.

Even though the Fumion AEI might not be the most suitable choice at high current densities, Fumion AEI in combination with Piperion AEM demonstrated repeatedly superior performances at lower current densities between 50 and 250 mA/cm^2^ (Figure 4b). Except for Piperion AEI/Sustainion AEM, Fumion AEI is the preferential choice for low current density operation.

The superior performance of the Piperion AEI at high current density can be further rationalized by the exceptional porosity retention in the catalyst layer. In fact, the BET surface area of the Pajarito Powder catalyst decreases by half in the Sustanion AEI and Fumion AEI-derived catalyst layers. On the contrary, Piperion AEI offers much higher retention of pores, particularly below 4 nm (Figure 5).

### 2.4 Effect of AEI on O_2_ Mass Transport in the Catalyst Layer

Due to the thickness of the Fe–N–C-based cathodes employed in this study (∼40 μm for 1.2 mg_Fe–N–C_ cm^–2^) compared to Pt/C (∼10 μm),^37^ O_2_ transport limitations are much more likely in Fe–N–C cathodes. As already observed during ORR measurements in the RDE setup, the diffusion-limiting currents in thin-film electrodes with AEIs are lower compared to Nafion, implying poor O_2_ permeability of the AEIs. The weight fraction of AEI in the catalyst layers was reduced for the GDE studies, in an attempt to limit the effect of excessive ionomer blockage on oxygen diffusion. Nevertheless, it is possible that gas-phase transport might be still impeded to a different degree depending on the AEI in the catalyst layer. Limiting current measurement enables the exploration of the effect of the AEI on the oxygen transport capabilities of the catalyst layer. For the sake of simplicity, the AEI is combined with its respective brand membrane (Fumion AEI/Fumasep AEM, Piperion AEI/Piperion AEM, Sustainion AEI/Sustainion AEM) in the set of experiments for the evaluation of the O_2_ mass transport.

Limiting current measurements is a widely applied technique to determine the oxygen transport resistance in MEAs in PEMFC.^42−44^ Nevertheless, the study of oxygen transport in AEMFC has been less explored. Only recently was a protocol developed to study the O_2_ mass transport in GDE setups.^45^ Increasing the ionomer to catalyst ratio for Nafion and Pt/C-based catalyst layers in an acidic environment led to voltage losses at high current densities for high ionomer to catalyst ratios due to oxygen transport limitations. The Fumion AEI-based catalyst layer has the highest mass transport resistance, followed by Sustainion AEI-based catalyst layers. (Figure 4h). The lowest mass transport resistance is measured for the Piperion AEI-based catalyst layer. Mass-transfer losses become dominant at high currents in the GDE ORR measurement. Therefore, the overpotentials at 2 A/cm^2^ are correlated with the mass transport resistance values. The overpotentials at high current densities measured with a concentration of 100% O_2_ in GDE follow the same trend as the *R*_T_ values (Figure 4h). We can conclude that the type of ionomer impacts the oxygen transport in the catalyst layer and thus the performance at current densities with high oxygen consumption.

### 2.5 Effect of the AEI on the Water Vapor Sorption Capabilities of Fe–N–C Catalyst Layers

As water is the reactant at the cathode in an AEMFC, cathodes are inclined to dry out at high current densities. Therefore, insights into the water sorption behavior of the catalyst layers and the interaction of water with the ionomer and pore structure are important to optimize the performance. The water sorption isotherms of all AEIs in the respective catalyst layer can be assigned to type 3 isotherms, with little sorption at low relative humidity and an exponential sorption at high relative humidity range (Figure 5c). Type 3 isotherms indicate that the strong adsorbate–adsorbate interactions of water molecules in the vapor dominate over the weak adsorbate–adsorbent interactions of water with the hydrophobic catalyst and ionomer.^46^ Below 40% relative humidity, the adsorption is controlled by hydrophilic functional groups in the catalyst layer.^47^ Therefore, the water uptake at low relative humidity can provide information regarding the hydrophobicity of the ionomers, which decreases in the order Fumion, Piperion, and Sustainion.

In the intermediate range of relative humidity (30 to 80% relative humidity), water molecules form multilayers around hydrophilic sites due to hydrogen bonding. When these localized clusters of water are saturated, they coalesce, which leads to water clusters and causes capillary condensations.^48^ The lower the relative pressure at which the rise begins, the smaller the capillaries as the onset of condensation shifts to higher pressure with increasing pore size. The upturn of water adsorption for Piperion AEI at a lower relative pressure compared to Fumion AEI is in agreement with the pore size distribution from the N_2_ sorption data (Figure 5b). In fact, the Piperion AEI catalyst layer has more mesopores with 3 nm pore width compared to the Fumion AEI-based catalyst layer.^49^ A steep isotherm at relative humidities higher than 80% implies that secondary pores are filled with water,^50^ which is pronounced for the Fumion AEI catalyst layer, which has the highest proportion of mesopores between 5 and 10 nm. Due to the water occupation of secondary pores in Fumion and Piperion AEI catalyst layers, these catalyst layers face a higher risk of blocking gas transport at high relative humidities. The similar hysteresis for all AEI/CLs points toward similar water retention capacities. After the relative humidity in the backward scan was reduced, Sustainion retained the highest amount of water compared to the other AEIs. The water adsorption isotherm of a catalyst layer with Pajarito Powder and QAPF-4 ionomer (wt %/wt % 0.43 I/C) has been reported with a similar type 3 isotherm.^51^ The water adsorption capacity of ∼470 mg_H_2_O_/g_cat_ (QAPF-4/Catalyst layer) compares to 471 mg_H_2_O_/g_cat_ (Fumion AEI/Catalyst layer), 466 mg_H_2_O_/g_cat_ (Piperion AEI/Catalyst layer), and 307 mg_H_2_O_/g_cat_ (Sustainion AEI/Catalyst layer).

### 2.6 Effect of the Alkaline Exchange Membrane on the ORR Overpotential in the GDE

**Figure 6:**
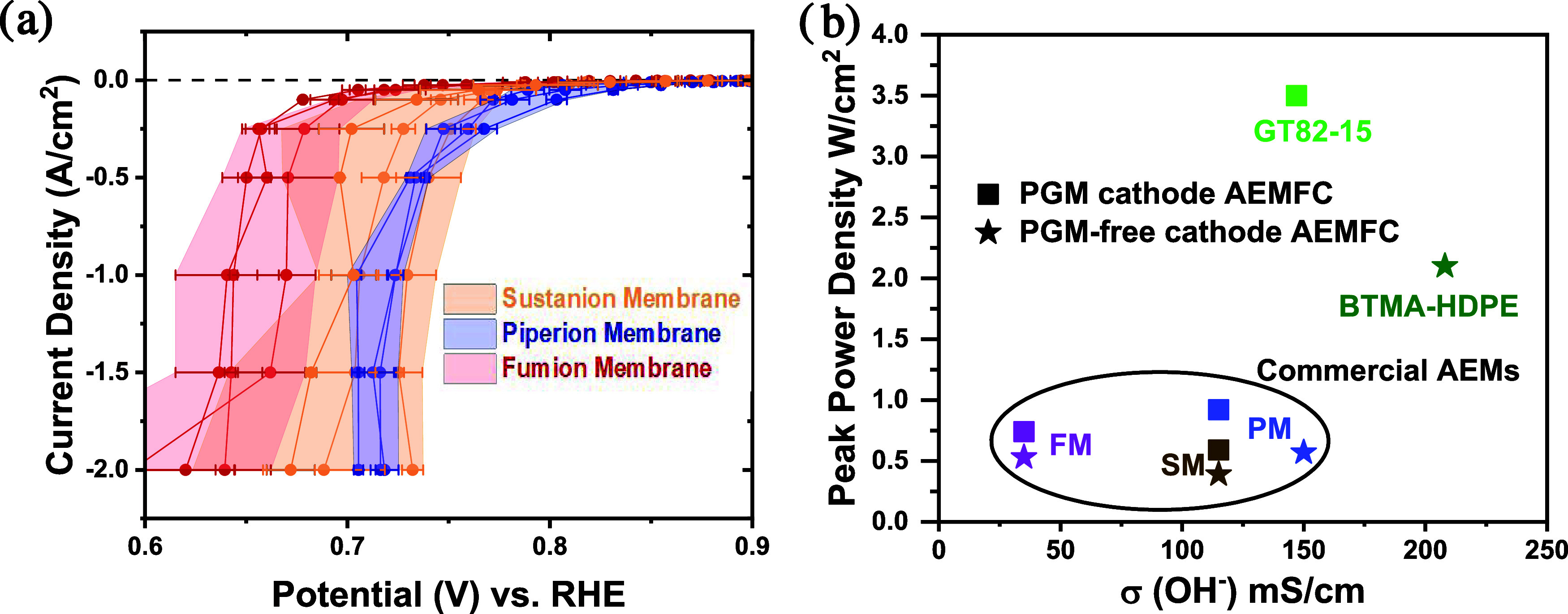
(a) Effect of the choice of the AEM membrane. The lines with same colors represent data collected with the same membrane and different AEI (red for Fumion, blue for Piperion, yellow for Sustanion), and the colored area shows the range of performance obtained with the same membrane. The GEIS protocol starts with galvanostatic steps and holding time from small current densities to high current densities in the order of −0.1 mA/cm^2^ (90 s), -1/-2.5/-5/-10 mA/cm^2^ (30 s), -25/-50/-10/-250 mA/cm^2^ (5 s), and -0.5/-1.0/-1.5/-2.0 A/cm^2^ (5 s) at an oxygen flow rate of 300 mL/min. Each single potential value is *iR*-compensated with the value of the uncompensated resistance corresponding to the magnitude of the impedance measured after the corresponding galvanostatic step. At least two sets of independent GEIS measurements for each type of sample are carried out. (b) Literature comparison of peak power densities versus hydroxide conductivities of commercial AEMs and best-performing AEMs in the best-reported AEMFCs with PGM-based (square) and PGM-free (star) cathodes.^6,13,52−60^(The testing conditions and references are listed in Table S5.)

To obtain a fair comparison between the different AEMs, membranes with similar thicknesses (between 40 and 55 μm) were chosen. At low and high current densities of the ORR, stark differences of 150 mV in overpotential are observed. The potentials range from 0.40 to 0.53 V at a current density of −0.05 A/cm^2^ and from 0.535 to 0.737 V at −2 A/cm^2^. By grouping the AEIs tested according to the membrane, it becomes evident that AEIs tested with Fumasep AEM in general display a higher overpotential (Figure 6a). It can also be observed that while all the electrodes tested with Piperion AEM offered similar performances, the choice of ionomer had a significant effect on the system based on Sustainion and Fumasep membranes.

To explain the differences in the ORR between the membranes, the hydroxide conductivities (σ(OH^–^)) of the respective membranes were analyzed. The σ(OH^–^) of Fumasep AEM at 25 °C has been reported to be 21 mS/cm at 25 °C, considerably lower than measured at temperatures relevant for AEMFC.^54^ On the contrary, the σ(OH^–^) of Sustainion AEM and Piperion AEM are reported to be 64 mS/cm at room temperature and 66 mS/cm at 25 °C,^55^ respectively. This trend of hydroxide conductivities correlates with the performance ranges of the respective AEMs in GDE ORR measurements. Other factors beyond the membrane properties could impact the interfacial resistance, including the adhesion of the catalyst layer to the membrane^56^ or the water transport at the interface between the catalyst layer and AEM.^46^ Therefore, to put the GDE studies in context with the AEMFC results, the commercial membranes at higher temperature in AEMFCs do not fall into the high-performance class of the research lab-produced AEMs for both PGM- and PGM-free cathode catalysts (Figure 6b, Table S5).^6,13,50−58^

## 3 Conclusions

We recommend that the RDE screening of catalysts in alkaline medium involve AEIs, so that they better resemble the MEA cathodes. We observed reduced oxygen permeability for all AEIs in comparison with Nafion. Currently, AEM manufacturers use the same composition for AEM and AEI, which means that they are geared to provide optimal AEM properties at the neglect of the AEI. We recommend that future improvements focus on providing distinct AEIs, optimized to provide high oxygen flux and permeability.^36,61^ The oxygen diffusion-limiting current in RDE is an easily accessible value to check the oxygen transport in newly developed AEIs. The RDE studies could be extended to quantify the mass transport based on parameters such as diffusion coefficient and solubility of O_2_ in the AEI.^62^ Due to reduced components compared to MEA, GDE half cells enable a testing platform to focus on the characterization of a single-catalyst layer with application-relevant loading and gas supply. Blending AEIs with the catalyst affects the mesopore and micropore structure of the catalyst material, with Fumion AEI and Sustainion AEI blocking the accessibility of micropores. While the lack of micropores might not be relevant at low current densities, at high current densities, the Piperion AEI-based catalyst layer with the highest BET surface area shows the best performance. Despite similar amounts of water present as a reactant in the Fumion AEI and Piperion AEI catalyst layers at 100% RH, the restricted oxygen transport capabilities limit the performance at high current densities for Fumion AEI. The hydration of the catalyst layer can be further optimized by tailoring the hydrophilic/hydrophobic properties via the polymer chemistry of the AEI.^63^ This study confirmed that the membrane’s hydroxide conductivity correlates with the performance of the AEM/AEI combination. Even though our study offers quantitative information regarding most of the vital parameters determining the performance of a catalyst layer, it should be noted that there are still aspects that cannot be independently characterized. One example is the interfacial properties between the ionomer and the membrane. These determine the continuity of the transport network for hydroxide ions, water and gases, the lack of which could be a limiting factor in the performance of the catalyst layer.^46,56^

Furthermore, as the chosen I/C ratio might not be optimal for each of the AEIs investigated, future work should focus on optimizing the I/C ratio^64,65^ in the catalyst layer for an individual AEI and also take the solvent composition^18,23^ into account for adjustment of the pore network of the catalyst layer. The ongoing developments in half-cell setups will enable us to study temperature^66^ and relative humidity effects on the cathode catalyst layer performance.

## Supporting Information Available

Molecular structure of the ionomers and membranes; experimental details for all the characterization techniques; experimental details for the electrochemical measurements; impedance spectra; XAS fitting results; GDE results; GEIS polarization measurements; limiting current measurements (PDF)
