# Supplementary material for: Influence of Commercial Ionomers and Membranes on a PGM-Free Catalyst in the Alkaline Oxygen Reduction
Source: ACS Appl Energy Mater. 2025 Mar 6;8(6):3470–80. doi: 10.1021/acsaem.4c02929 (PMC11938204; doi:10.1021/acsaem.4c02929)
Supplement: Supplementary file 1 — ae4c02929_si_001.pdf [file ae4c02929_si_001.pdf]

**Supporting Information**

# The influence of commercial Ionomers and Membranes on a PGM-free catalyst in the Alkaline Oxygen Reduction

*Simon Kellner [a], Ziyang Liu [a], Francesco D'Acierno [a], Angus Pedersen [a], Jesús Barrio [a], Sandrine Heutz [b], Ifan E. L. Stephens [b], Silvia Favero [a*], Maria-Magdalena Titirici [a,c*]*

[a] Department of Chemical Engineering, Imperial College London, London SW7 2AZ, United Kingdom

[b] Department of Materials, Royal School of Mines, Imperial College London, London SW7 2AZ, United Kingdom

[c] Advanced Institute for Materials Research (WPI-AIMR) Tohoku University 2-1-1 Katahira, Aobaku, Sendai, Miyagi 980-8577, Japan

* silvia.favero@icn2.cat ; m.titirici@imperial.ac.uk

## 1. Introduction

**Figure S1.** The molecular structures of commercial anion exchange ionomers (AEIs).

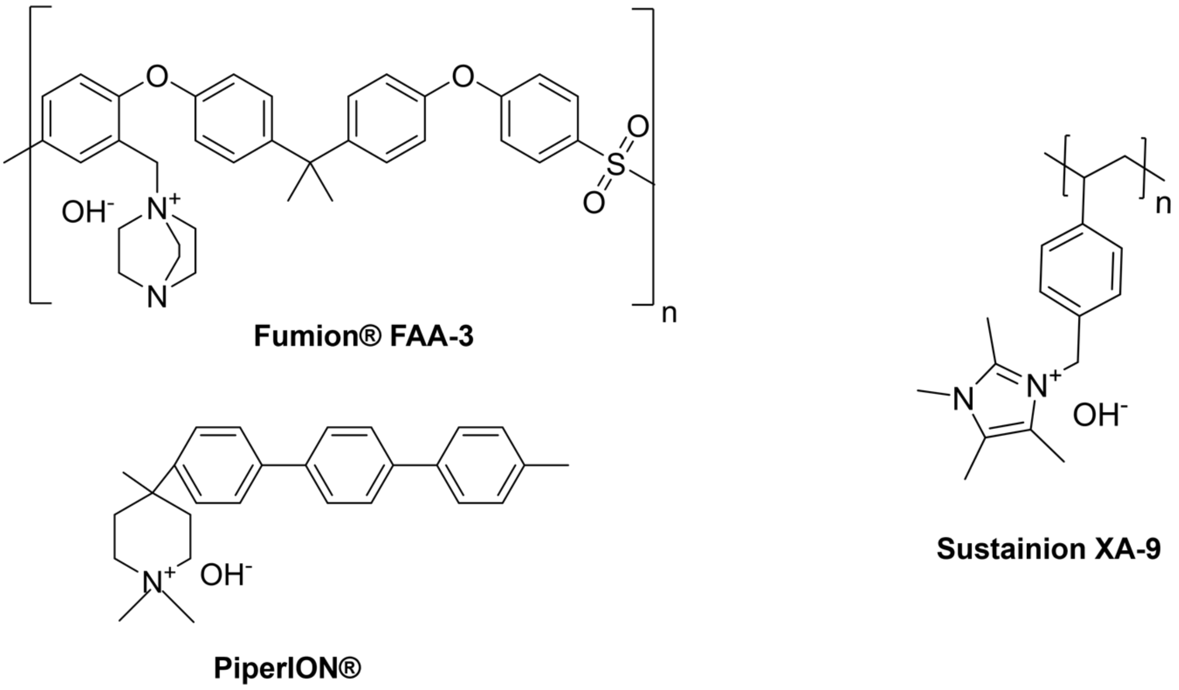

**Table S1.** PGM-based cathodes in AEMFC with peak power densities (PPD) > 2.0 W/cm$^2$.

| Reference | AEM / AEI | Anode / Cathode | PPD | Operating conditions |
|---|---|---|---|---|
| 1 | AEM: BTMA-HDPE<br>AEI: BTMA-ETFE<br>BTMA = Benzyltrimethylammonium<br>ETFE = Poly(ethylene-coptetrafluoroethylene)<br>HDPE = high density polyethylene | Anode: PtRu/C<br>(0.4 $mg_{Pt}/cm^2$)<br>Cathode: Pt/C<br>(0.4 $mg_{Pt}/cm^2$) | 2.5 W/cm$^2$ | 80°C ($H_2$-$O_2$)<br>92% RH<br>Flow rate: 1 L/min<br>No back pressure |
| 2 | AEM: PTFE-reinforced poly(norborene) GT64-15<br>AEI: BTMA-ETFE | Anode: PtRu/C<br>(0.7 mg/cm$^2$)<br>Cathode: Pt/C<br>(0.6 mg/cm$^2$) | 3.4 W/cm$^2$ | 80°C ($H_2$-$O_2$)<br>Anode / Cathode dew points:<br>67°C / 74°C |
| 3 | AEM: GT82-15*<br>AEI: ETFE-g-poly(VBTMAC)<br>*Poly(norborene)-based | Anode: PtRu/C<br>(0.7m mg/cm$^2$)<br>Cathode: Pt/C<br>(0.6 mg/cm$^2$) | 3.5 W/cm$^2$ | 80°C ($H_2$-$O_2$)<br>Anode / Cathode dew points:<br>66°C / 75°C<br>Anode / Cathode back pressure:<br>0.05 MPa / 0.1 MPa |
| 4 | AEM: GT64-15*<br>AEI: Anode: GT78*/ Cathode: GT32*<br>*Poly(norborene)-based | Anode: PtRu/C<br>(0.7 mg/cm$^2$)<br>Cathode: Pt/C<br>(0.6 mg/cm$^2$) | 3.2 W/cm$^2$ | 80°C ($H_2$-$O_2$) |
| 5 | AEM: PFBP-14<br>AEI: PFTP-13<br>PFBP = Poly(fluorene N,N-dimethylpipiridinium-co-nonfluoride)<br>PFTP = Poly(fluorne-co-terphenyl N,N'-dimethylpiperidinium) | Anode: PtRu/C<br>(0.42 mg/cm$^2$)<br>Cathode: Pt/C<br>(0.33 mg/cm$^2$) | 2.3 W/cm$^2$ | 80°C ($H_2$-$O_2$)<br>Flow rate: 1 L/min<br>Anode / Cathode RH:<br>75% / 100% |
| 6 | AEM: PDTP-25<br>AEI: PFBP<br>PDTP = Poly(diphenyl-terphenylpiperidinium) | Anode: PtRu/C<br>(0.39 mg/cm$^2$)<br>Cathode: Pt/C<br>(0.26 mg/cm$^2$) | 2.6 W/cm$^2$ | 80°C ($H_2$-$O_2$)<br>Anode / Cathode RH:<br>75% / 100%<br>Anode / Cathode back pressure:<br>1.3 bar / 1.3 bar |
| 7 | AEM: b-PTP-2.5**<br>AEI: PFBP<br>PFBP = Poly(fluorenyl-co-biphenyl piperidinium)<br>**Branched poly(aryl piperidinium) | Anode: PtRu/C<br>(0.6 mg/cm$^2$)<br>Cathode:<br>(0.4 mg/cm$^2$) | 2.3 W/cm$^2$ | 80°C ($H_2$-$O_2$)<br>Anode / Cathode RH:<br>75% / 100%<br>Anode / Cathode back pressure:<br>1.3 bar / 1.3 bar |
| 8 | AEM: b-PDTP-Trip-5***<br>AEI: b-PFBP-Trip-3.5***<br>***Triptycene-branched poly(arylpiperidinium) | Anode: PtRu/C<br>(0.39 mg/cm$^2$)<br>Cathode: Pt/C<br>(0.26 mg/cm$^2$) | 2.5 W/cm$^2$ | 80°C ($H_2$-$O_2$)<br>Anode / Cathode RH:<br>75% / 100%<br>Anode / Cathode back pressure:<br>1.3 bar / 1.3 bar |

## Table S2: Fe-N-C-based cathodes in AEMFC with PPD > 1.0 W/cm$^2$

| Reference | AEM / AEI | Anode / Cathode | PPD | Operating conditions |
|---|---|---|---|---|
| 9 | AEM: BTMA-HDPE<br>AEI: BTMA-ETFE | Anode: PtRu/C<br>(0.6 mg/cm$^2$)<br>Cathode: Fe-N-C<br>Pajarito Powder<br>(1 mg/cm$^2$) | 2.1 W/cm$^2$ | 80°C ($H_2$-$O_2$)<br>Anode / Cathode dew points:<br>70°C / 75°C<br>Anode / Cathode back pressure:<br>200 kPa |
| 10 | AEM: no information<br>AEI: BTMA-ETFE | Anode: PtRu/C<br>(0.6 mg/cm$^2$)<br>Cathode: Fe-N-C<br>$Fe_{0.5}$-$NH_3$<br>(0.9 mg/cm$^2$) | 1.4 W/cm$^2$ | 65°C ($H_2$-$O_2$)<br>Anode / Cathode dew points:<br>55°C / 60°C |
| 11 | AEM: BTMA-HDPE<br>AEI: BTMA-ETFE | Anode: PtRu/C<br>(0.6 mg/cm$^2$)<br>Cathode: Fe-N-C<br>$Fe_{0.5}$-dry<br>(0.91 mg/cm$^2$) | 1.8 W/cm$^2$ | 80°C ($H_2$-$O_2$)<br>Anode / Cathode dew points:<br>71°C / 77°C<br>Anode / Cathode back pressure:<br>0.05 MPa / 0.1 MPa |
| 12 | AEM: PVA-0.8PQVBC40%<br>AEI: Nafion | Anode: PtRu/C<br>(0.3 mg/cm$^2$)<br>Cathode: Fe-N-C<br>$FeN_x$-CNTs<br>(4.0 mg/cm$^2$) | 1.2 W/cm$^2$ | 60°C ($H_2$-$O_2$)<br>Anode / Cathode back pressure<br>2 bar / 2 bar |
| 13 | AEM: PVA-1.8PVBMP<br>AEI: QPPO<br>QPPO = quaternized poly(2,6-dimethyl-1,4-phenylene) oxide | Anode: PtRu/C<br>(0.4 mg/cm$^2$)<br>Cathode: Fe-N-C<br>$FeN_x$CNTs<br>(2 mg/cm$^2$) | 1.2 W/cm$^2$ | 60°C ($H_2$-$O_2$)<br>Flow rate: 400 mL/min<br>Anode / Cathode back pressure<br>0.1 MPa / 0.1 MPa |
| 14 | AEM: Xion Pention-72-15CL<br>AEI: Pention D35 | Anode: PtRu/C<br>(0.6 $mg_{Cat}$/cm$^2$)<br>Cathode: Fe-N-C<br>Fe-N-PDC-HA<br>(1 mg/cm$^2$) | 1.1 W/cm$^2$ | 80°C ($H_2$-$O_2$)<br>Anode / Cathode dew points:<br>71°C / 77°C<br>Anode / Cathode flow rate:<br>500 mL/min / 1000 mL/min<br>Cathode back pressure 0.1 MPa |
| 15 | AEM: Poly(norobene) tetra block copolymer<br>AEI: Poly(norobene) tetra block copolymer | Anode: CST-PtRu/NC<br>(0.05 mg/cm$^2$)<br>Cathode: Fe-N-C<br>Pajarito Powder<br>(1.0 mg/cm$^2$) | 1.2 W/cm$^2$ | 80°C ($H_2$-$O_2$)<br>Anode / Cathode dew points:<br>70°C / 75°C |

**Experimental**

## 2. Experimental Section

### 2.1. Materials

The commercially available products are used without modification. Fe-N-C catalyst: Pajarito Powder PMFD14401 (Abbreviation: Pajarito Powder or Fe-N-C).All AEI and AEM materials are supplied from Fuel Cell Store (USA):

Ionomers:

Fumasep® Fumion FAA-3-SOLUT-10 (10wt% in NMP) (Abbreviation: FI)

PiperION® Anion Exchange Dispersion (5 wt% in ethanol) (Abbreviation: PI)

Sustainion® XA-9 Alkaline Ionomer (5 wt% in ethanol) (Abbreviation: SI)

Nafion™ D-521 (5 wt% in $H_2O$ and isopropanol)

Nafion™ D-1021 (10 wt% in $H_2O$)

Membranes:

Fumasep® FAA-3-50: 45-55 µm (dry thickness) (Abbreviation: FM)

PiperION® AEM: 40 µm (dry thickness) (Abbreviation: PM)

Sustainion® X37-50 Grade 60: 50µm (dry thickness) (Abbreviation: SM)

## 2.2. Ink properties measurements

**Ultra-small angle X-ray scattering (USAXS)**

The USAXS/SAXS study of particle and agglomerate size for dispersed RDE catalyst inks (with ink formulation described in Electrochemical Measurement part) was conducted at BL11 – BCD-SWEET beamline at ALBA synchrotron. Freshly sonicated ink samples are transferred into a glass capillary tube (1 mm diameter) with the help of syringe and sealed with an epoxy resin for transport. Before the measurement the capillary tubes are immersed into an ultrasonic bath for 10 minutes. The background scattering data from the glass capillary tube filled with the solvent solutions (Ethanol/$H_2O$) were recorded and subtracted from the scattering data for each sample. The samples were exposed to a monochromatic X-ray beam in the operational energy range from 6.5 to 20 keV (including WAXS). The scattered X-ray intensity was measured with a Pilatus3 S 1M detector. The X-ray intensity data were collected in scattering angle ranges from $4x10^{-3}$ $A^{-1}$ to 0.75 $A^{-1}$ (SAXS), 0.4 $A^{-1}$ to 10.8 $A^{-1}$ (WAXS). The scattering data were analyzed in the data analysis software SASView with simulations of scattering fitting functions.

**Rheology**

The shear stress and viscosity data were measured with an Anton Paar MCR 302 Rheometer. A volume of 20 mL of the GDE inks (with ink formulation described in Electrochemical Measurement part), reference ink of Pajarito Powder with same concentration as in GDE inks in ethanol and ethanol only are poured into the rotational cylinder cup and temperature is adjusted to 25°C. The data is collected at steady state stepping the shear rate from 1 to 2510 $s^{-1}$ with logarithmic spacing.

**Dynamic light scattering (DLS)**

DLS measurements on the GDE inks (with ink formulation described in Electrochemical Measurement part) were performed using a Malvern Zetasizer MicroV. Before the measurements, the inks were diluted by factor 100. The analyzed inks contained 0.02 mg/mL catalyst and 0.005 mg/mL ionomer. The inks are sonicated in the quartz cuvette for 10 minutes before the measurement. The program carried out 60 runs for 10s each and is repeated three times.

## 2.3. Electron microscopy

The GDE samples are cut with a razor blade to prepare the cross-section samples. Cross-section samples are stuck with carbon tape on a stub with a vertical edge with the cross-section of the sample facing to the top. The top-view samples are prepared with carbon tape on top of a regular stub. A film of chromium (40 nm) is sputtered onto the specimen. The images are taken on a Zeiss Leo Gemini 1525.

## 2.4. Sorption measurements

GDE inks (with ink formulation described in Electrochemical Measurement part) were sprayed manually with an airbrush set (Paasche) powered by an Iwata Smart Jet Pro Airbrush compressor onto aluminum foil. The CL covers an area of 20 $cm^2$ with a loading of 1.0 $mg_{Fe\text{-}N\text{-}C}/cm^2$ and the weight of loading is determined by pre- and post-spray weighing. Spraying onto aluminum foil has the advantage over catalyst coated membranes (CCM) of eliminating the contribution of the membrane on the measurement of gas and water sorption.[16]

**Nitrogen sorption**

Nitrogen sorption experiments of the CLs on Al foil were conducted on a 3Flex (Micromeritics) adsorption analyzer at 77 K within a relative pressure range from $10^{-5}$ to 0.99 $p/p_0$. Before the measurement, the samples were degassed at $10^{-6}$ bar, 453 K for 24 hours, using the micromeritics VacPrep. The BET surface area was obtained with the linearized BET equation, in the region where Roqouerol plot increases monotonically.[17] The pore size distribution was calculated using slit geometry and heterogeneous surface-2D-NLDFT model in-built with 3Flex software.

**Water vapor sorption**

Water sorption isotherms of the CLs on Al foil were recorded on a 3Flex (Micromeritics) adsorption analyzer at 298 K within a relative pressure range from $1^{-3}$ to 0.86 $p/p_0$. Before the measurements the CLs are dried at 453 K for 24 hours in vacuum and the water was purified by performing three freeze-thaw cycles to remove any air and dissolved gases.

## 2.5. Electrochemical Measurements

### Rotating Disc Electrode

Electrochemical characterization was performed in a standard three-electrode cell using a potentiostat Multi AUTOLAB/M101 (Metrohm). The working electrode was a glassy carbon disk (Ø = 5 mm). Inks contained 4 mg of the Fe-N-C catalyst, 480 µg ethanol (99.5%, Sigma Aldrich), 480 µg 18.2 MΩ deionized water and 40 µg of ionomer dispersion (Nafion™, or Piperion, or Sustainion) or 20 µg of ionomer dispersion (Fumion). The choice of the ionomer / catalyst weight ratio of 0.5 is consistent with literature[18,19], corresponding to 33wt%/66wt% I/C. After sonication in ice bath for 30 minutes, the ink is dropcasted (10 µL) on a freshly polished circular area glassy carbon disk to load 200 $\mu g_{Fe\text{-}N\text{-}C}/cm^2$. The ink was dried under rotation (300 rpm). A glassy carbon rod served as counter electrode. A Hg/HgO (30 mm, Redox.me) reference electrode was calibrated against a $H_2$ (1 bar) purged Pt RDE tip (Ø = 3 mm, Metrohm) working electrode (1000 rpm) and platinum rod counter electrode in 0.1 M KOH (99.995% Suprapur).[20] After purging $N_2$ (99.9998%, Ultrapure Plus), 50 cyclic voltammograms were recorded at 50 mV/s and 1600 rpm in the potential range of 0.20 $V_{RHE}$ to 1.05 $V_{RHE}$ to precondition the catalyst. Subsequently, 3 cyclic voltammograms were recorded at 10 mV/s and 0 rpm in the potential range of 0.20 $V_{RHE}$ to 1.10 $V_{RHE}$ to evaluate the capacitive current. After purging $O_2$ (99.99998% BIP® Plus) for 15 minutes, three cyclic voltammograms at 10 mV/s and 1600 rpm were recorded in the potential range from 0.25 $V_{RHE}$ to 1.10 $V_{RHE}$. The pseudocapacitance was corrected by subtracting the current obtained previously in $N_2$ from the cyclic voltammograms in $O_2$. The ohmic drop was calculated for each measurement by means of electrochemical impedance measurements from $10^5$ to $10^{-1}$ Hz at 0.85 V at 1600 rpm by taking the first intercept of the real impedance axis in the Nyquist plot.

The kinetic current densities ($j_{kin}$) were calculated at 0.85 $V_{RHE}$ using the geometric disk current density ($j_d$) at 0.85 $V_{RHE}$ and the limiting current density ($j_{lim}$) at 0.25 $V_{RHE}$ following equation (1):

$$j_{kin} = \frac{j_d \cdot j_{lim}}{j_d - j_{lim}} \quad (1)$$

The mass activity (MA) can be calculated with the following equation (2):

$$MA = \frac{j_{kin}}{Catalyst\ Loading} \quad (2)$$

**Gas Diffusion Electrode Half Cell**

Electrochemical characterization was performed in an updated small gas-diffusion-electrode[21,23] (GDE) cell using a potentiostat PGSTAT204 with FRA32M Module (Metrohm) in combination with a BOOSTER10A. A typical ink composition for the GDE was 20wt% Ionomer / 80wt% Fe-N-C catalyst with a catalyst content of 2 $mg_{Fe\text{-}N\text{-}C}$/mL. All the ionomers were tested with the same ink formulation, deposition technique and testing conditions. Of course, the optimal ink formulation is likely to be different for all the ionomers, and better performance could be achieved with an individual optimization of the ink formulation of each of them. Nevertheless, it was decided to test the ionomers with the same formulation, to allow to correlate more easily the ionomer structure, with the morphology and performance of the catalyst layer. An ionomer loading of 20% was selected for all the ionomers, following separate reports on different AEIs reporting this value as optimal.[22]

The detailed ink formulation contained 10 mg Fe-N-C (Pajarito Powder PMFD14401) and 50.7 µL AEI (Sustainion (5 wt% in ethanol), or Piperion (5 wt% in ethanol)) or 19.4 µL AEI (Fumion (10 wt% in NMP). The reference sample contained 10 mg Fe-N-C , 25 µL Nafion™ D-1021 (10 wt% in $H_2O$) and the solvents 2.475 mL DI $H_2O$ and 2.5 mL isopropanol (99.5% Honeywell™). The inks are subjected to sonication in an ice bath for 1 hour, and are stirred overnight. The GDEs are prepared via an Exactacoat (Sonotek) ultrasonic spraycoater with a 48 kHz nozzle at 0.15 mL/min (air shaping 0.3 psi) with an offset serpentine spray pattern on a 5 $cm^2$ gas diffusion layer (Freudenberg H23C8) while heated at 40°C on a vacuum plate. The gas diffusion layer is weighed pre and post-cathode spraying to determine the cathode catalyst loading. For the working electrode preparation, a piece (Ø = 3 mm, Boehm) is cut out of the GDE, and inserted into a hole (Ø = 3 mm, Boehm) of a H23C8 GDL (Freudenberg). The AEM membrane (Ø = 20 mm) is positioned between the upper cell body and the H23C8 GDL with its hydrophobic coating facing the AEM and with the GDE catalyst layer facing the membrane

exactly located underneath the hole in the upper cell body. The lower cell body with a flow-field facing towards the H23C8 GDL is connected to a bubbler and gas flow meter (Bronkhorst) with a flow rate of 300 mL/min unless stated otherwise. _The bubbler was used to humidify the gas before entering the GDE cell The counter electrode was a platinum coil. The hydrogen reference electrode (RHE) was manufactured in-house and the hydrogen in the capillary is prepared before each single sample. The compartment of the RHE is contact with the AEM via a luggin capillary. The upper cell compartment of the GDE was filled with as 1 M aqueous KOH (99.995% Suprapur). The measurement protocols with the GDE half-cell are described into detail in Table S3 and S4.

**Membrane activation**

The membranes are stored and pre-treated as recommended by the respective manufacturer.

Fumasep FAA-3-50: The membrane is delivered in bromide form ($Br^-$) and dry form. Prior to the GDE application, the membrane is converted into the $OH^-$ by treating it in aqueous 1.0 M KOH (99.995% Suprapur) for 24 hours at room temperature in a sealed PET bottle.[23]

PiperION® AEM: The membrane is delivered in the bicarbonate form. Prior to the GDE application, the membrane is placed into an aqueous solution of 0.5 M KOH (99.995% Suprapur) for 1 hour at room temperature. Then, it is transferred to a fresh aqueous solution of 0.5 M KOH (99.995% Suprapur) for an additional 1 hour at room temperature. Subsequently, the membrane is rinses with DI water, and transferred into fresh 0.5 M KOH (99.995% Suprapur) solution in a sealed PET bottle.[24]

Sustainion® X37-50 Grade 60: The membrane arrived soaked in a ethylene glycol plasticizer to prevent cracking. Prior to the GDE application, the membrane is activated for 3 hours in 1 M KOH at 55°C, and then transferred into a fresh 1.0 M (99.995% Suprapur) solution in a sealed PET bottle.[25]

## Cleaning procedure

Before each experiment the upper cell compartment is boiled two times in a glass beaker with 18.2 MΩ deionized water and rinsed with 18.2 MΩ deionized water. To avoid contamination, the cell is stored in 18.2 MΩ deionized water in a sealed glass container between the experiments. When changing to a new catalyst layer composition, the cell is soaked in 95% $H_2SO_4$ with Nochromix® overnight, then extensively rinsed in 18.2 MΩ deionized water before being transferred into a beaker for the boiling procedure.

## Electrochemical Protocol

For each data GEIS polarization curve at least two samples are tested. The electrochemical protocol for the GDE ORR testing is adapted from the community standard protocol.[26] The last data point of each galvanostatic step is iR-compensated by taking a EIS at each galvanostatic step. The value for uncompensated resistance is the magnitude of the impedance, for which the phase angle is closest to 0 in the high frequency region > 1000 Hz. Typically the solution resistance was below 1 Ω.

**Table S3.** Electrochemical protocol for the GDE ORR testing

| | Step | Electrochemical Technique | | Parameters |
|---|---|---|---|---|
| **1** | Preconditioning ($N_2$) | Cyclovoltammograms | Gas purge (Flow rate)<br>Purge duration<br>Potential limits<br>Scan rate<br>Number of cycles | $N_2$ (300 mL/min)<br>15 min<br>0.06 – 1.1 V vs. RHE<br>50 mV/s, 500 mV/s<br>10 each |
| **2** | Polarization Curve ($O_2$) | Galvanostatic steps coupled with impedance spectroscopy (GEIS) | Gas purge (Flow rate)<br>Purge duration<br>Current steps (hold time)<br><br><br><br>EIS frequency range<br>iR-compensation | $O_2$ (300 mL/min)<br>10 min<br>- 0.1 mA/cm$^2$ (90 s),<br>- 1/-2.5/-5/-10 mA/cm$^2$ (30 s),<br>- 25/-50/-10/-250 mA/cm$^2$ (5 s)<br>- 0.5/-1.0/-1.5/-2.0 A/cm$^2$ (5 s)<br>f = 10 kHz – 1 Hz<br>100% post-correction |

The electrochemical protocol for the GDE $O_2$ mass transport measurements is adapted from an established protocol.[27] For each type of CL at least 2 samples are tested. A concentration of 2.5 vol% $O_2$ were mixed into $N_2$. The total flow is set to 500 mL/min. Galvanostatic steps of 20 mA/cm$^2$ in 5 seconds were carried out. At a certain current, all oxygen in the catalyst layer has reacted, concomitant with a sudden potential jump. Corresponding to the definition of the limiting current, changes in potential do not change the current any further ($\partial i/\partial E = 0$).[28] In this study, the limiting current is determined as the value of the current step before the potential jump. (Figure Using the limiting current $i_{lim}$, the mass transport resistance $R_T$ can be calculated:

$$R_T = \frac{4\,F\,x_0^{dry-in}}{i_{lim}} \frac{p - p_w}{RT} \quad \text{(Eq. S1)}$$

with the Faraday constant F = 96485 C/mol, the concentration of $O_2$ regulated to the dry mole fraction $x_0^{dry-in}$, p denotes total gas pressure which is atm in the small GDE setup, $p_w$ is the water vapor pressure, T is the absolute cell temperature and R is the universal gas constant.

The average value of the mass transport resistance $R_T$ and error is reported.

**Table S4. Electrochemical protocol for the $O_2$ mass transport measurements**

| | Step | Electrochemical Technique | | Parameters |
|---|---|---|---|---|
| **1** | Oxygen Mass Transport (2.5% $O_2$) | Galvanostatic Staircase with Electrochemical Impedance Spectroscopy | Gas purge composition | 2.5% $O_2$ / 97.5% $N_2$ |
| | | | Gas purge flowrates | 12 mL/min $O_2$ / 488 mL/min $N_2$ |
| | | | Total flowrate | 500 mL/min |
| | | | Purge duration | 15 min |
| | | | Scan rate | 20 mA/cm$^2$ / 5 s = 5 mAcm$^{-2}$s$^{-1}$ |
| | | | EIS frequency | f = 5 kHz |

**Figure S2.** EIS in RDE at 0.85 V in oxygen saturated 0.1 M KOH at 1600 rpm.

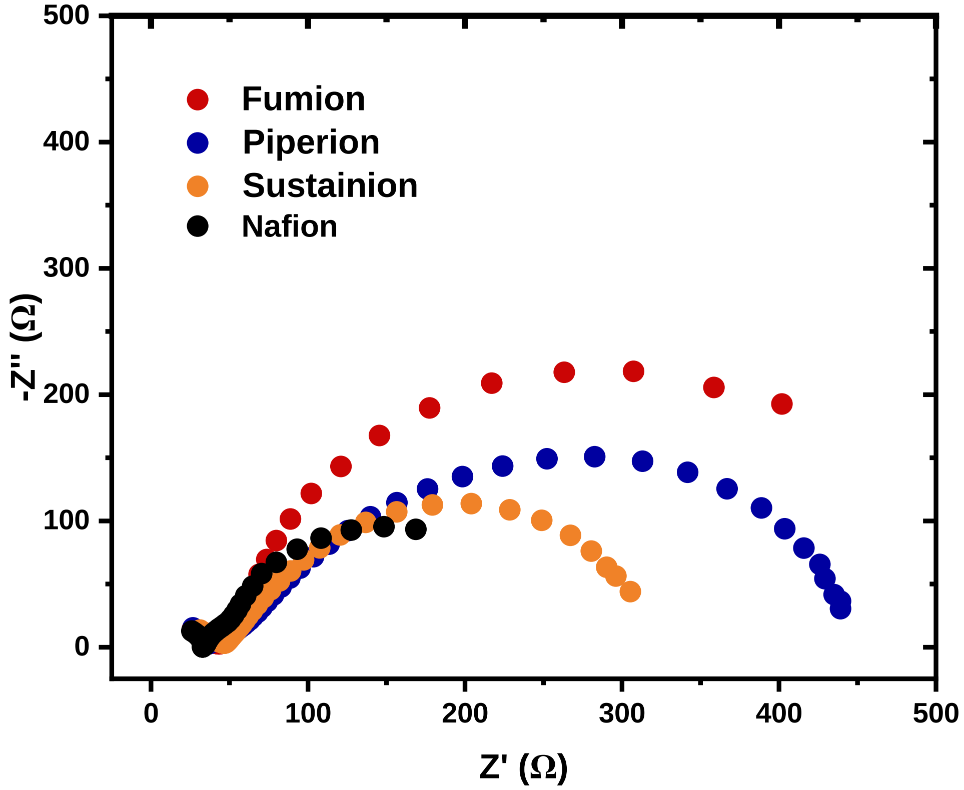

**Figure S3.** SAXS fitting of a) Fumion ink, b) Piperion ink, c) Sustainion ink, d) Nafion ink.

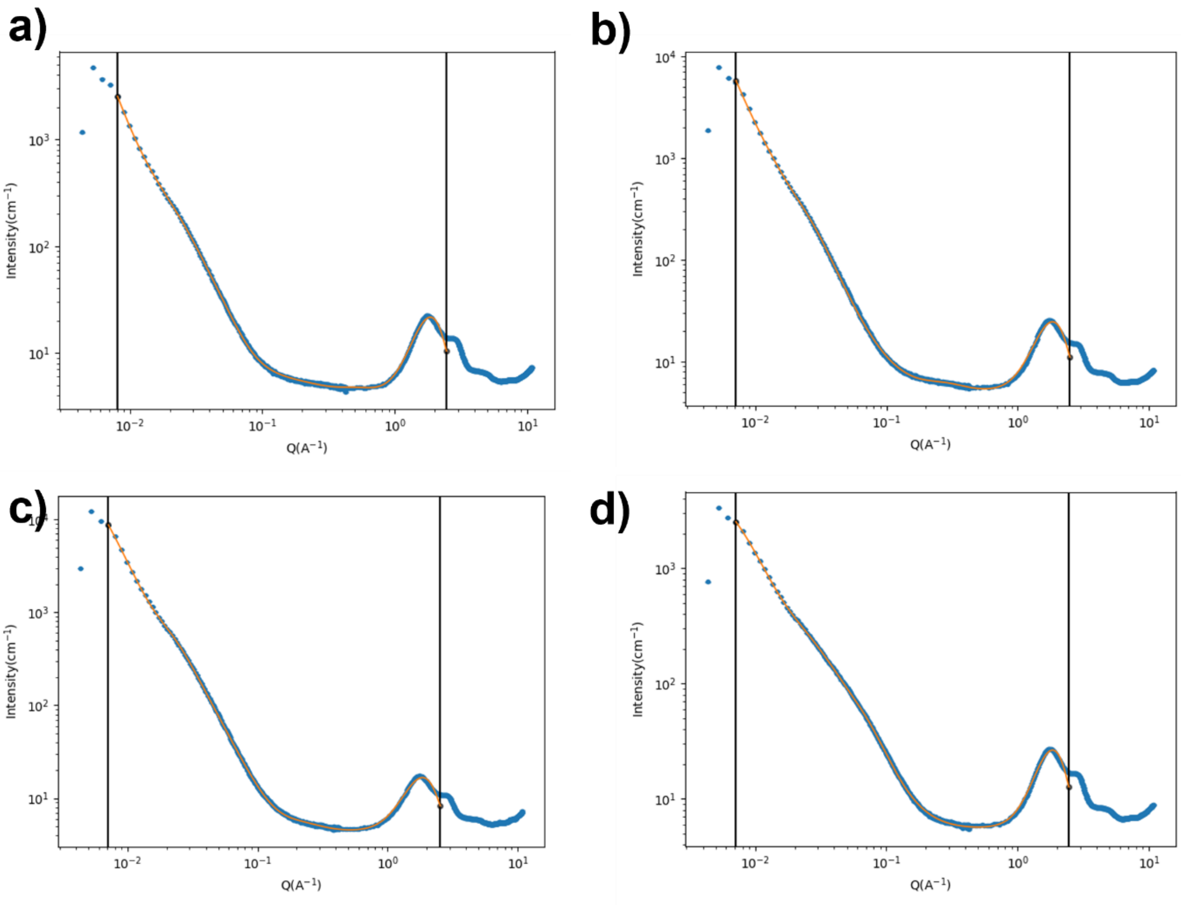


**Figure S4.** The macroscopic images of the sprayed samples for $N_2$ sorption experiments.

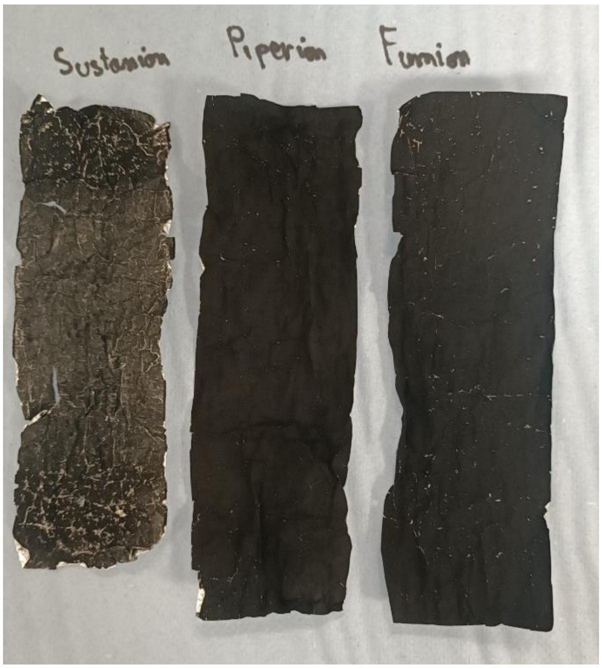


**Figure S5.** ORR GEIS polarization curves of anionomers and Nafion combined with SM membrane.

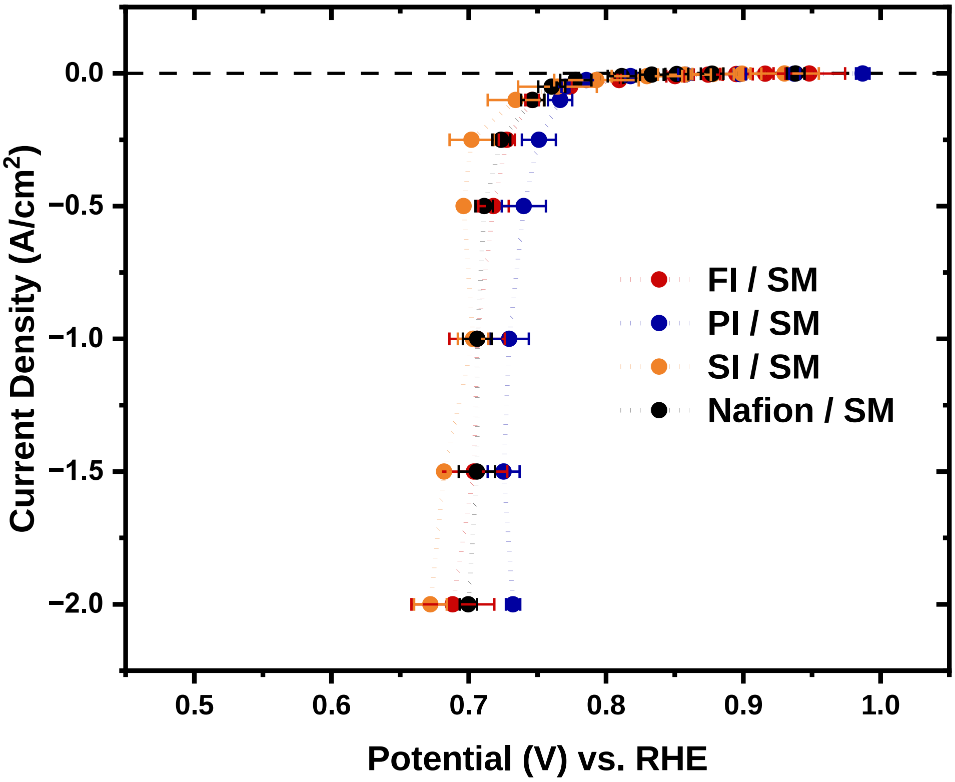

**Figure S6** Tafel plot for the GEIS polarization measurements

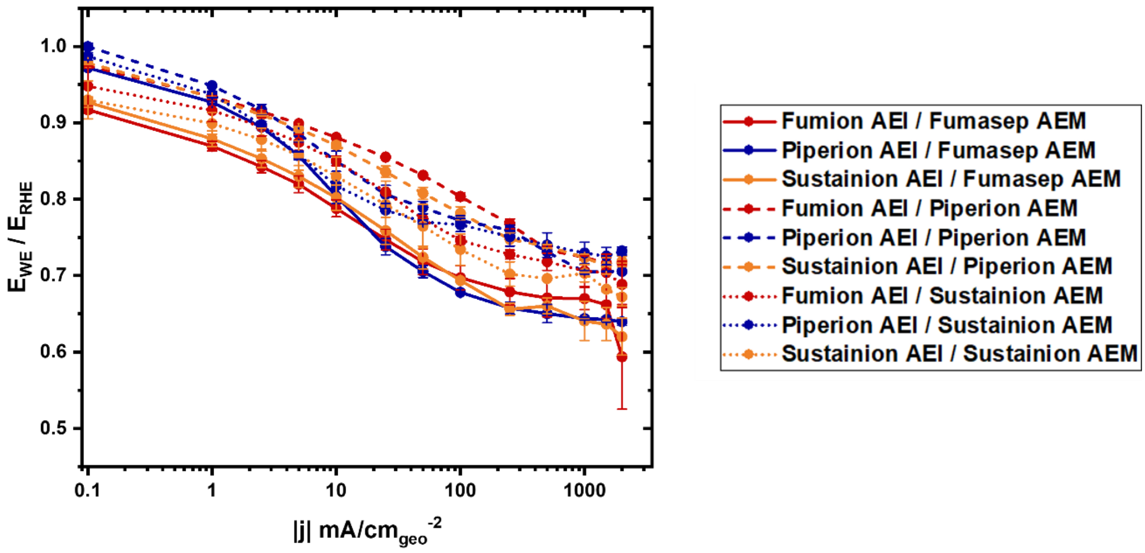

**Figure S7.** Examplary limiting current measurements for two catalyst layer samples of Fumion AEI based catalyst layers at 2.5% $O_2$.

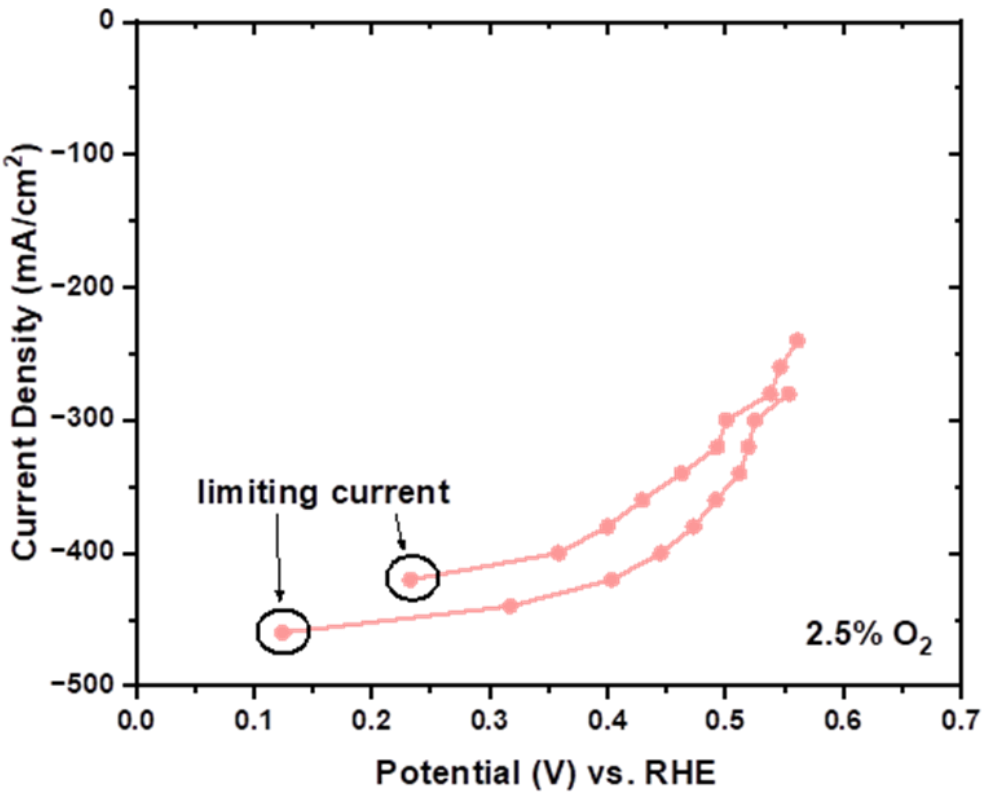

**Table S5.** Commercial AEMs vs. lab-scale AEMs: PPDs and hydroxide conductivities σ($OH^-$).

| Reference | AEM / AEI | Anode / Cathode | PPD/ σ($OH^-$) | Operating conditions |
|---|---|---|---|---|
| 29<br>30 | AEM: FAA-3-50<br>AEI: FAA-3 | PGM<br>Anode: PtRu/C<br>(0.4 mg/cm$^2$)<br>Cathode: Pt/C<br>(0.4 mg/cm$^2$) | PPD: 0.74 W/cm$^2$<br>σ($OH^-$): 35 mS/cm<br>(T = 60°C) | 60°C ($H_2$-$O_2$)<br>Anode: 0.8 L/min /<br>Cathode: 1.0 L/min |
| 31<br>29 | AEM: FAA-3-50<br>AEI: FAA-3 | PGM-free cathode<br>Anode: Pt/C<br>(0.5 mg$_{Pt}$/cm$^2$)<br>Cathode: FeNC-4000<br>(0.5 mg/cm$^2$) | PPD: 0.53 W/cm$^2$<br>σ($OH^-$): 35 mS/cm<br>(T = 60°C) | 60°C ($H_2$-$O_2$)<br>Anode: 0.8 L/min /<br>Cathode: 1.0 L/min<br>Anode / Cathode RH:<br>80% / 90%<br>Back pressure: 1.5 bar |
| 32<br>33 | AEM: Sustainion<br>X37-50<br>AEI: Sustainion | PGM<br>Anode: Pt/C<br>(0.76 mg$_{Pt}$/cm$^2$)<br>Cathode: Pt/C<br>(0.76 mg$_{Pt}$/cm$^2$) | PPD: 0.59 W/cm$^2$<br>σ($OH^-$): 115 mS/cm<br>(T = 60°C) | 60°C ($H_2$-$O_2$)<br>Anode: 500 sccm<br>Cathode: 1000 sccm |
| 34<br>33 | AEM: Sustainion<br>X37-50 Grade RT<br>AEI: Sustainion | PGM-free cathode<br>Anode: Pt/C<br>(0.76 mg$_{Pt}$/cm$^2$)<br>Cathode: FeCo-N-C<br>(0.70 mg/cm$^2$) | PPD:0.39 W/cm$^2$<br>σ($OH^-$): 115 mS/cm<br>(T = 60°C) | 60°C ($H_2$-$O_2$)<br>Anode: 500 sccm<br>Cathode: 1000 sccm<br>Anode / Cathode RH:<br>93% / 93% |
| 35<br>36 | AEM: PiperION<br>AEI: PiperION | PGM<br>Anode: PtRu/C<br>(0.4 mg/cm$^2$)<br>Cathode: Pt/C<br>(0.4 mg/cm$^2$) | PPD: 0.92 W/cm$^2$<br>σ($OH^-$): 115 mS/cm<br>(T = 60°C) | 60°C ($H_2$-$O_2$)<br>Anode: 1000 sccm<br>Cathode: 1000 sccm |
| 37<br>36 | AEM: PiperION<br>AEI: PiperION | PGM-free cathode<br>Anode: PtRu/C<br>(0.4 mg/cm$^2$)<br>Cathode: Co/Fe-N-C<br>(2mg/cm$^2$) | PPD: 0.57 W/cm$^2$<br>σ($OH^-$): 150 mS/cm<br>(T = 80°C) | 80°C ($H_2$-$O_2$)<br>Anode: 0.2 L/min /<br>Cathode: 0.4 L/min<br>Anode / Cathode RH:<br>70% / 100%<br>Back pressure: 100 kPa |
| 3 | AEM: GT82-15*<br>AEI: ETFE-g-<br>poly(VBTMAC)<br>*Poly(norborene)-<br>based | Anode: PtRu/C<br>(0.7m mg/cm$^2$)<br>Cathode: Pt/C<br>(0.6 mg/cm$^2$) | PPD: 3.5 W/cm$^2$<br>σ($OH^-$): 147 mS/cm<br>(T = 80°C) | 80°C ($H_2$-$O_2$)<br>Anode / Cathode dew points:<br>66°C / 75°C<br>Anode / Cathode back pressure:<br>0.05 MPa / 0.1 MPa |
| 9 | AEM: BTMA-HDPE<br>AEI: BTMA-ETFE | PGM-free cathode<br>Anode: PtRu/C<br>(0.6 mg/cm$^2$)<br>Cathode: Fe-N-C<br>Pajarito Powder<br>(1 mg/cm$^2$) | PPD: 2.1 W/cm$^2$<br>σ($OH^-$): 208 mS/cm<br>(T = 80°C) | 80°C ($H_2$-$O_2$)<br>Anode / Cathode dew points:<br>70°C / 75°C<br>Anode / Cathode back pressure:<br>200 kPa |

## References

1. Wang, K.; Peng, X.; Mustain, W. E.; Varcoe, J. R. Radiation-grafted anion-exchange membranes: the switch from low- to high-density polyethylene leads to remarkably enhanced fuel cell performance. *Energy Environ. Sci.* **2019**, 12, 1575-1579

2. Huang, G.; Mandal, M.; Peng, X.; Yang-Neyerlin, A. C.; Pivovar, B. S.; Mustain, W. E.; Kohl, P. A. Composite Poly(norbornene) Anion Conducting Membranes for Achieving Durability, Water Management and High Power (3.4 W/cm2) in Hydrogen/Oxygen Alkaline Fuel Cells. *J. Electrochem. Soc.* **2019**, 166, F637

3. Mandal, M.; Huang, G.; Ul Hassan, N.; Peng, X.; Gu, T.; Brooks-Starks, A. H.; Bahar, B.; Mustain, W. E.; Kohl, P. A. The Importance of Water Transport in High Conductivity and High-Power Alkaline Fuel Cells. *J. Electrochem. Soc.* **2020**, 167, 054501

4. Ul Hassan, N.; Mandal, M.; Huang, G.; Firouzjaie, H. A.; Kohl, P. A.; Mustain, W. E. Achieving High-Performance and 2000 h Stability in Anion Exchange Membrane Fuel Cells by Manipulating Ionomer Properties and Electrode Optimization. *Adv. Energy Mater.* **2020**, 10, 2001986

5. Chen, N.; Wang, H. H.; Kim, S. P.; Kim, H. M.; Lee, W. H.; Hu, C.; Bae, J. Y.; Sim, E. S.; Chung, Y.-C.; Jang, J.-J.; Yoo, S. J.; Zhuang, Y.; Lee, Y. M. Poly(fluorenyl aryl piperidinium) membranes and ionomers for anion exchange membrane fuel cells. *Nat. Commun.* **2021**, 12, 2367

6. Chen, N.; Hu, C.; Wang, H. H.; Kim, S. P.; Kim, H. M.; Lee, W. H.; Bae, J. Y.; Park, J. H.; Lee, Y. M. Poly(Alkyl-Terphenyl Piperidinium) Ionomers and Membranes with an Outstanding Alkaline-Membrane Fuel-Cell Performance of 2.58 Wcm$^{-2}$. *Angew. Chem. Int. Ed.* **2021**, 60, 7710-7718

7. Wu, X.; Chen, N.; Klok, H.-A.; Lee, Y. M.; Hu, X. Branched Poly(Aryl Piperidinium) Membranes for Anion-Exchange Membrane Fuel Cells. *Angew. Chem. Int. Ed.* **2022**, 61, e202114892

8. Hu, C.; Kang, N. Y.; Kang, H. W.; Lee, J. Y.; Zhang, X.; Lee, Y. J.; Jung, S. W.; Park, J. H.; Kim, M.-G.; Yoo, S. J.; Lee, S. Y.; Park, C. H.; Lee, Y. M. Triptycene Branched Poly (aryl-co-aryl piperidinium) Electrolytes for Alkaline Anion Exchange Membrane Fuel Cells and Water Electrolyzers. *Angew. Chem. Int. Ed.* **2024**, 63, e202316697

9. Adabi, H., Shakouri, A.; Ul Hassan, N.; Varcoe, J. R.; Zulevi, B.; Serov, A.; Regalbuto, J. R.; Mustain, W. E.. High-performance commercial Fe-N-C cathode electrocatalyst for anion-exchange membrane fuel cells. *Nat. Energy* **2021**, 6, 834-843

10. Firouzjaie, H. A.; Mustain, W. E. Catalytic Advantages, Challenges, and Priorities in Alkaline Membrane Fuel Cells. *ACS Catal.* **2020**, 10 (1), 225-234

11. Adabi, H.; Santori, P. G.; Shakouri, A.; Peng, X.; Yassin, K.; Rasin, I. G.; Brandon, S.; Dekel, D. R.; Ul Hassan, N.; Sougrati, M.-T.; Zitolo, A.; Varcoe, J. R.; Regalbuto, J. R.; Jaouen, F.; Mustain, W. E. Understanding how single-atom site density drives the performance and durability of PGM-free Fe-N-C cathodes in anion exchange membrane fuel cells. *Mater. Today Adv.* **2021**, 12, 100179

12. He, Q.; Zeng, L.; Wang, J.; Jiang, J.; Zhang, L.; Wang, J.; Ding, W.; Wei, Z. Polymer-coating-induced synthesis of $FeN_x$ enriched carbon nanotubes as cathode that exceeds 1.0Wcm$^{-2}$ peak power in both proton and anion exchange membrane fuel cells. *J. Power Sources* **2021**, 489, 229499

13. Zeng, L.; He, Q.; Liao, Y.; Kuang, S.; Wang, J.; Ding, W.; Liao, Q.; Wei, Z. Anion Exchange Membrane Based on Interpenetrating Polymer Network with Ultrahigh Ion Conductivity and Excellent Stability for Alkaline Fuel Cell. *AAAS* **2020**, 2020, 4794706

14. Teppor, P.; Jäger, R.; Koppel, M.; Volobujeva, O.; Palm, R.; Månsson, M.; Härk, E.; Kuchovski, Z.; Aruväli, J.; Kooser, K.; Granroth, S.; Käämbre, T. J. Nerut, E. Lust. Unlocking the porosity of Fe-N-C catalysts using hydroxyapatite as a hard template en route to eco-friendly high-performance AEMFCs. *J. Power Sources* **2024**, 591, 233816

15. Adabi, H.; Shakouri, A.; Zitolo, A.; Asset, T.; Khan, A.; Bohannon, J.; Chattot, R.; Williams, C.; F. Jaouen, F.; Regalbuto, J. R.; Mustain, W. E. Multi-atom Pt and PtRu catalysts for high performance AEMFCs with ultra-low PGM content. *Appl. Cat. B: Environ.* **2023**, 325, 122375

16. Kusoglu, A.; Kwong, A.; Clark, K. T.; Gunterman H. P.; Weber, A. Z. Water Uptake of Fuel-Cell Catalyst Layers. *J. Electrochem. Soc.* **2012**, 159, F520-F535

17. Rouquerol, J.; Rouquerol, F.; Sing, K. S. W.; Llewellyn, P., G. Maurin G. "Adsorption by powders and porous solids: principles." Methodology and Applications (Academic, New York) **2014**

18. Santori, P. G.; Speck, F. D.; Li, J.; Zitolo, A.; Jia, Q.; Mukerjee, S.; Cherevko, S.; Jaouen, F. Effect of Pyrolysis Atmosphere and Electrolyte pH on the Oxygen Reduction Activity, Stability and Spectroscopic Signature of $FeN_x$ Moieties in Fe-NC Catalysts. *J. Electrochem. Soc.* **2019**, 166, F3311-F3320

19. Daems, N.; Breugelsman, T.; Vankelecom, I. F. J.; Pescarmona, P. P. Influence of the Composition and Preparation of the Rotating Disk Electrode on the Performance of Mesoporous Electrocatalysts in the Alkaline Oxygen Reduction Reaction. *ChemElectroChem* **2018**, 5, 119-128

20. Kawashima, K.; Márquez, R. A.; Son, Y. J.; Guo, C.; Vaidyula, R. R.; Smith, L. A.; Chukwuneke, C. E.; Mullins, C. B.Accurate Potentials of Hg/HgO Electrodes: Practical Parameters for Reporting Alkaline Water Electrolysis. *ACS Catal.* **2023**, 13, 1893-1898

21. Wiberg, G. K. H.; Nösberger, S.; Arenz, M. Evolution of a GDE setup: Beyond ambient conditions. *Curr. Opin. Electrochem.* **2022**, 36, 101129

[22] Z. Yang, R. Guo, R. Malpass-Evans, M. Carta, N. B. Mckeown, M. D. Guiver, L. Wu, T. Xu, Angew. Chem., Int. Ed. 2016, **55**, 11499.

J.i E. Park, S. Y. Kang, S.-H. Oh, J. K. Kim, M. S.u Lim, C.-Y. Ahn, Y.-H. Cho, Y.-E. Sung, Electrochim. Acta 2019, **295**, 99.

R. B. Kaspar, M. P. Letterio, J. A. Wittkopf, K.e Gong, S. Gu, Y. Yan, J. Electrochem. Soc. 2015, **162**, F483.

23. https://www.fuelcellstore.com/spec-sheets/fumapem-faa-3-50-technical-specifications.pdf

24. https://www.fuelcellstore.com/versogen-piperion-aem-mechanically-reinforced-15um-73800004

25. https://dioxidematerials.com/product/sustainion-anion-exchange-membrane/

26. Ehelebe, K; Schmitt, N.; Sievers, G.; Jensen, A. W.; Hrnjić, A.; Jiménez, P. C.; Kaiser, P.; Geuß, M.; Ku, Y.-P.; Jovanovič, P.; Mayrhofer, K. J. J.; Etzold, B.; Hodnik, N.; Escudero-Escribano, M.; Arenz. M.; Cherevko, S. Benchmarking Fuel Cell Electrocatalysts Using Gas Diffusion Electrodes: Inter-lab Comparison and Best Practices. *ACS Energy Lett.* **2022**, 7 (2) , 816-826

27. Lauf, P.; Lloret, V.; Geuß, M.; Collados, C. C.; Thommes, M.; Mayrhofer, K. J. J.; Ehelebe, K.; Cherevko, S. Characterization of Oxygen and Ion Mass Transport Resistance in Fuel Cell Catalyst Layers in Gas Diffusion Electrode Setups. *J. Electrochem. Soc.* **2023**, 170 (6), 064509

28. Beuscher, U. Experimental method to determine the mass transport resistance of a polymer electrolyte fuel cell. *J. Electrochem. Soc.* **2006**, 153 (9), A1788

29. Cha, M. S.; Park, J. E.; Kim, S.; Han, S.H.; Shin, S. H.; Yang, S. H.; Kim T. H.; Yu, D. M.; So, S.; Hong, Y. T.; Yoon, S. J.; Oh, S. G.; Kang, S. Y.; Kim, O. H.; Park, H. S.; Bae, B.; Sung,

Y. E.; Cho, Y. E.; Lee, J. Y. Poly(carbazaole)-based anion-conducting materials with high performance and durability for energy conversion devices. *Energy Environ. Sci.* **2020**, 13, 3633-3645

30. Zhegur-Khais, A.; Kubannek, F.; Krewer, U.; Dekel, D. R. Measuring the true hydroxide conductivity of anion exchange membranes. *J. Membr. Sc.* **2020**, 612, 118461

31. Kim, M.-J.; Kim, S.; Park, J. E.; Hwang, C.-C.; Lee, S.; Kang, S. Y.; Jung, D.; Cho, Y.-H.; Kim, J.; Lee, K.-S.; Sung, Y.-E. Controlling active sites of Fe-N-C electrocatalysts for oxygen electrocatalysis. *Nano Energy* **2020**, 78, 105395

32. Calderon, J.; Huq, N.; Zheng, M.; Zhou, Y. Power Enhancement of Anion Exchange Membrane Fuel Cells (AEMFCs) Through Spray-Coating of Membrane With Partially Reduced Graphene Oxide. *J. Undergrad. Chem. Eng. Res.* **2022**, 75

33. Liu, Z.; Sajjad, S. D.; Gao, Y.; Yang, H.; Kaczur, J. J.; Masel, R. I. Int. J. Hydrog. Energy, The effect of membrane on an alkaline water electrolyzer. *Int. J. Hydrog. Energy* **2017,** 42, 29661-29665

34. Raut, A.; Fang, H.; Lin, Y.-C.; Fu, S.; Sprouster, D.; Shimogawa, R.; Frenkel, A. I.; Bae, C.; Douglin, J. C.; Lillojad, J.; Tammeveski, K.; Zeng, Z.; Bliznakov, S.; Rafailovich, M.; Dekel, D. R. Migration and Precipitation of Platinum in Anion-Exchange Membrane Fuel Cells. *Angew. Chem. Int. Ed.* **2023**, 62, e202306754

35. Hyun, J.; Lee, D. W.; Oh, E.; Bae, H.; Park, J.; Doo, G.; Kim, H.-T. Manufacturing and structural control of slurry-cast catalyst layers for AEMFC. *J. Power Sources* **2023**, 573, 233161

36. Chae, J. E. ; Choi, J. ; Lee, S. ; Park, C. ; Kim, S. Effects of fabrication parameters of membrane-electrode assembly for high-performance anion exchange membrane fuel cells. *J. Ind. Eng. Chem.* **2023**, 133, 255-262

37. Pei, Y.; Zhu, W.; Yue, R.; Yao, J.; Liu, X.; Wang, L.; Zhang, J.; Yin, Y.; Guiver, M. D. Fine adjustment of catalyst agglomerate for the controllable construction of Co/Fe-N-C catalyst layers. *J. Power Sources* **2023**, 566, 232904
